# Graph databases in systems biology: a systematic review

**DOI:** 10.1093/bib/bbae561

**Published:** 2024-11-20

**Authors:** Ilya Mazein, Adrien Rougny, Alexander Mazein, Ron Henkel, Lea Gütebier, Lea Michaelis, Marek Ostaszewski, Reinhard Schneider, Venkata Satagopam, Lars Juhl Jensen, Dagmar Waltemath, Judith A H Wodke, Irina Balaur

**Affiliations:** Medical Informatics Laboratory, University Medicine Greifswald, Walther-Rathenau-Straße 48, Greifswald 17475, Germany; Luxembourg Centre for Systems Biology, University of Luxembourg, 6 Avenue du Swing, Belvaux L-4367, Luxembourg; Luxembourg Centre for Systems Biology, University of Luxembourg, 6 Avenue du Swing, Belvaux L-4367, Luxembourg; Medical Informatics Laboratory, University Medicine Greifswald, Walther-Rathenau-Straße 48, Greifswald 17475, Germany; Medical Informatics Laboratory, University Medicine Greifswald, Walther-Rathenau-Straße 48, Greifswald 17475, Germany; Medical Informatics Laboratory, University Medicine Greifswald, Walther-Rathenau-Straße 48, Greifswald 17475, Germany; Luxembourg Centre for Systems Biology, University of Luxembourg, 6 Avenue du Swing, Belvaux L-4367, Luxembourg; Luxembourg Centre for Systems Biology, University of Luxembourg, 6 Avenue du Swing, Belvaux L-4367, Luxembourg; Luxembourg Centre for Systems Biology, University of Luxembourg, 6 Avenue du Swing, Belvaux L-4367, Luxembourg; Department of Veterinary and Animal Sciences, Faculty of Health and Medical Sciences, University of Copenhagen, Grønnegårdsvej 15, 1870 Frederiksberg C, Denmark; Medical Informatics Laboratory, University Medicine Greifswald, Walther-Rathenau-Straße 48, Greifswald 17475, Germany; Medical Informatics Laboratory, University Medicine Greifswald, Walther-Rathenau-Straße 48, Greifswald 17475, Germany; Luxembourg Centre for Systems Biology, University of Luxembourg, 6 Avenue du Swing, Belvaux L-4367, Luxembourg

**Keywords:** graph databases, RDF, NoSQL databases, systems biology, network biology, ontology

## Abstract

Graph databases are becoming increasingly popular across scientific disciplines, being highly suitable for storing and connecting complex heterogeneous data. In systems biology, they are used as a backend solution for biological data repositories, ontologies, networks, pathways, and knowledge graph databases. In this review, we analyse all publications using or mentioning graph databases retrieved from PubMed and PubMed Central full-text search, focusing on the top 16 available graph databases, Publications are categorized according to their domain and application, focusing on pathway and network biology and relevant ontologies and tools. We detail different approaches and highlight the advantages of outstanding resources, such as UniProtKB, Disease Ontology, and Reactome, which provide graph-based solutions. We discuss ongoing efforts of the systems biology community to standardize and harmonize knowledge graph creation and the maintenance of integrated resources. Outlining prospects, including the use of graph databases as a way of communication between biological data repositories, we conclude that efficient design, querying, and maintenance of graph databases will be key for knowledge generation in systems biology and other research fields with heterogeneous data.

## Introduction

In the last decade, new technologies and approaches emerged to extract large amounts of biological data, to interconnect data types across biological layers (proteins, metabolites, pathways, drugs, etc.) and to capture complex data relationships such as drug–biomarker–disease. Traditional approaches of storing biological data in a tabular format using relational databases present shortcomings when integrating biological content that is diverse, complex, and highly connected [1]. Such data are important for systems biology [2], where biological processes are studied by assembling and modelling the entirety of relevant knowledge. This requires efficient exploration of highly connected and heterogeneous data and their inter-relationships [3].

Graph databases (GDBs) have become popular for data integration, exploration, and visualization in systems biology due to their potential to overcome the limitations of the relational approach [1, 4, 5]. Graphs can naturally integrate and represent interactions between heterogeneous biological entities in the form of so-called knowledge graphs (KGs), allowing for efficient data traversal and exploration without the need to join multitudes of tables, a computationally expensive task [1, 4]. GDBs are particularly efficient for querying highly interconnected data such as pathway data [1, 6, 7], where execution performance for complex queries on gene-related paths and relationships between proteins is greatly improved using a GDB solution [7].

Here, we provide a systematic review on the application of GDBs in systems biology. We focus on the problems addressed by the GDB methodology, on identified solutions, and their advantages and limitations. We also discuss approaches towards harmonized KGs. Finally, we review current needs and new research questions in systems biology and related domains in the context of GDBs.

The review focuses on the top 16 available GDB technologies (db-engines.com/en/ranking/graph+dbms) including but not limited to ArangoDB, Neo4j, OrientDB, and Virtuoso. Initially, we automatically extracted a set of 681 publications on GDB applications in systems biology with a cut-off date of 31 March 2023. Each of the abstracts was then manually and independently annotated by two reviewers to assess relevance, applicability, documentation, and sustainability for further inclusion in this review. Finally, a list of 179 publications was considered for the review. Code developed for automatic publication metadata extraction and the manual annotations for each publication are available at github.com/ilyamazein/gdbreview. Details on the protocol including the inclusion and exclusion criteria are provided in the Methods section.

In the Background section, we briefly introduce relational and graph databases. In the Results section, we present examples of GDB applicability with a focus on (i) pathway biology, (ii) relevant ontologies, and (iii) relevant tools and analytical methods, as well as provide examples from COVID-19 research. These three sections follow the logic of these three questions: what is the content, how are the data structured, and how are the data analysed and used? In the Discussion section, we address challenges and future prospects of applying GDB technologies in the biological domain, and we conclude by outlining advantages of GDB usage.

## Background

### Relational databases

Relational databases are well established and widely used for storing and querying biological data [8]. They are founded on the concept of tables (or relations). A table represents a type of entity. The columns represent named attributes of the entity, and the rows represent instances of the entity itself. Each row of a table should be identified by a unique key (formed by one or more attributes, usually a unique ID attribute) called its primary key. A relational database may be queried using a query language, usually SQL (Structured Query Language). For complex relations, intermediate tables are generated. However, this also results in potentially complex queries.

Relational databases offer many algorithms for the efficient retrieval of bulk structured data [9]. However, they work best with data in a suitable, uniform structure, namely, nonsparsely populated and well-defined tables. When presented with highly connected, sparsely populated, or heterogeneous data, a relational database becomes less efficient. Specifically, the time and computational resources required to complete complex queries involving several joins among multiple tables increase considerably, thus making exploration of interconnected data challenging [1, 6].

### Graph databases

A graph database (GDB) represents data and their inter-relationships using a graph, where an object or concept can be represented as a node and a relationship between two objects as an edge. Notably, GDBs are schema-optional: the representation of objects and relationships in the graph is not necessarily determined by a schema, does not require an initial normalization step, and can be adapted without the need to restructure the database itself [5, 10].

GDBs are particularly efficient for storing and querying highly connected data such as pathway data or for performing traversal queries [1, 6]. While it is possible to implement graph algorithms in relational databases, it typically requires complex SQL queries and multiple join operations to traverse relationships stored in tables. For example, representing a graph structure in a relational database involves creating tables for nodes and edges and using foreign keys to establish relationships. Subgraph mining and other graph algorithms necessitate repeated joining of these tables to explore paths and connections, which can be computationally expensive and slow, particularly for large and highly interconnected datasets.

In systems biology, GDBs, which rely on graph representation, can naturally integrate and represent heterogeneous biological entities as networks allowing for efficient data traversal exploration without the need to join multiple tables [1, 4]. Moreover, graphs provide a more natural solution for human visualization and interpretation, whereas the relational model is more suitable for computer interpretation, making it hard to visualize data in a way people can quickly and easily understand [11]. For example, in the GDB implementation of Reactome, the average time for pathway query was reduced by 93% in comparison with the relational database implementation [6]. In another example of comparing Neo4j and MySQL performance on a variety of queries exploring gene-related paths and relationships between proteins, authors reported that the Neo4j-based implementation outperformed the MySQL solution for all queries and highlighted that the difference was more evident (reaching a magnitude of 7 with respect to measured time performance) between the two systems when the queries became more complex [7].

The two most frequent graph models are ‘Resource Description Framework (RDF) triple stores’ (w3.org/TR/rdf-concepts) and ‘labelled property graphs (LPGs)’ [5].

The ‘RDF model’ is an open World Wide Web Consortium (W3C) standard used to describe resources and relationships between them in the form of triples (w3.org/TR/2004/REC-rdf-concepts-20040210). A triple is composed of three elements: a subject, an object, and a predicate that describes the relationship between them (see Supplementary Fig. S1.A for an example). Each element of a triple is generally denoted using an Internationalized Resource Identifier, such as a URL. A set of triples forms an RDF graph, where resources are nodes and relationships are edges between these nodes. RDF stores are typically queried using SPARQL (SPARQL Protocol and RDF Query Language) (w3.org/TR/rdf-sparql-query), which is a declarative language that aims to be similar to SQL.

The ‘LPG model’ enriches the base graph structure with additional features: (i) nodes may have one or more labels that indicate their type(s); (ii) edges must have one type; and (iii) both edges and nodes may have a set of properties defined as key-value pairs (see Supplementary Fig. S1B for an example). Currently, one of the most popular LPG database management platforms in systems biology is Neo4j (neo4j.com), which has its own declarative language, (entitled Cypher), and presents intuitive exploration and visualization features that facilitate its usability.

While RDF databases are better suited to publish and exchange structured data representations, LPGs are more efficient when it comes to schema complexity, graph density, and querying the data itself [12]. Therefore, if the KGs demand sharing data in an interoperable way (e.g. ontologies), the RDF would be a better option, while, for KGs that require efficient analysis and storage, (e.g. biological pathways), an LPG would be better suited for the job.

### List of graph databases

In Table 1, we provide the details on the data model, initial release, licence type, and the number of associated publications from PubMed (pubmed.ncbi.nlm.nih.gov) or PubMed Central (PMC; ncbi.nlm.nih.gov/pmc) for the top GDB technologies (both open-source and commercial) as reported in the DB-Engines resource (db-engines.com/en, reference date September 2023). Additionally, there are publications that offer a more detailed comparison of specific graph database technologies, as well as their experimental evaluation [13, 14].

**Table 1:** Ranking of the top 16 graph open-source and commercial databases based on DB-Engines (db-engines.com/en/ranking/graph+dbms, reference date September 2023), an initiative to collect and present information on database management systems. We include the number of articles found in PMC that use or mention these databases.

#	Graph database name	Database model	Initial release	Licence	PMC^a^	Rank
1	Neo4j^b^	Graph	2007	Community Edition: GPLv3	544	50.39
2	Microsoft Azure Cosmos DB	Multimodel	2014	Commercial	1	35.45
3	Virtuoso^b^	Multimodel	1998	Open Source Edition: GPLv2	69	5.38
4	OrientDB^b^	Multimodel	2010	Community Edition: Apache 2	35	4.33
5	ArangoDB^b^	Multimodel	2012	Free Edition: Apache 2	25	4.29
6	Memgraph	Graph	2017	Commercial	1	2.88
7	GraphDB	Multimodel	2000	Commercial	18	2.6
8	Amazon Neptune	Multimodel	2017	Commercial	2	2.54
9	JanusGraph^c^	Graph	2017	Apache 2	7	2.39
10	Nebula Graph^c^	Graph	2019	Apache 2	141	2.33
11	Stardog	Multimodel	2010	Commercial	6	2.28
12	TigerGraph	Graph	2017	Commercial	5	2.21
13	Dgraph^c^	Graph	2016	Apache 2	6	1.89
14	Fauna	Multimodel	2014	Commercial	4	1.69
15	Giraph^c^	Graph	2013	Apache 2	4	1.65
16	AllegroGraph^b^	Multimodel	2013	Commercial; Free edition	36	1.15

a This column is based on authors’ analysis for the number of hits in PMC publications, last updated in September 2023.

b Commercial with open source or free version available.

c Open source.

## Results

**Figure 1:**
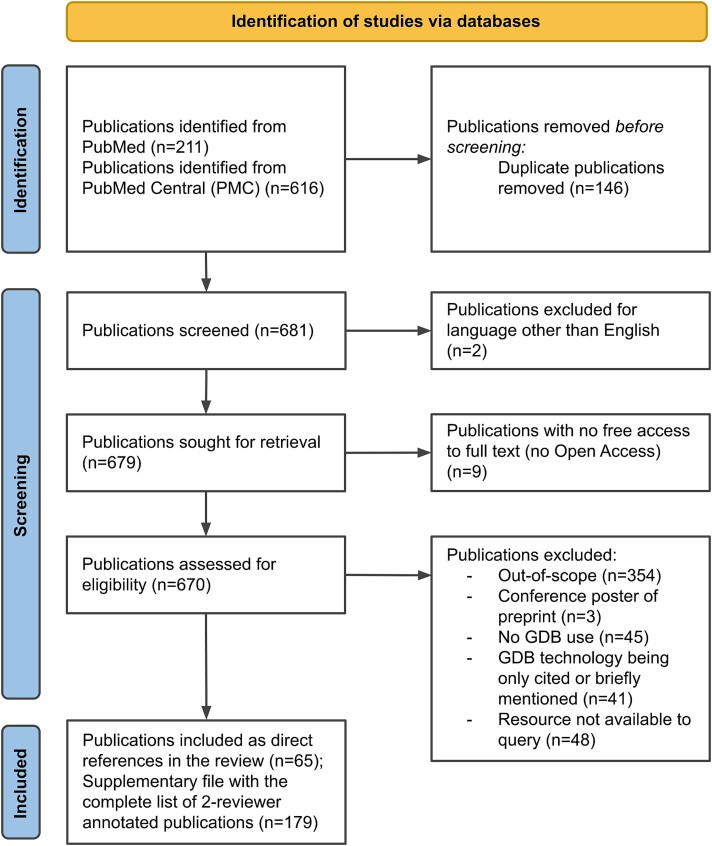
PRISMA 2020 flow diagram for our review, which included searches of PubMed and PMC databases.

We selected an initial set of 681 publications related to GDBs by querying PubMed and PMC (see Methods for more details). We then annotated, classified, and evaluated all publications of this initial set manually (two reviewers per publication, seven main categories) and selected a set of 179 publications as suitable for this review. The seven main categories (reviews, methods, software, primary resources, integrated resources, ontologies, and other) were initially chosen as a technical classification for the publications and then narrowed down according to the focus of the review (see Supplementary Tables S1 and S2 for the number of selected publications per category and section of this review, respectively). The workflow for publication selection is shown in Fig. 1, which follows the PRISMA 2020 approach for systematic review reporting (Preferred Reporting Items for Systematic Reviews and Meta-Analyses—PRISMA: prisma-statement.org) [15]).

Notably, the number of publications in biology mentioning at least one GDB published each year is increasing (see Supplementary Fig. S2). Throughout the selected publications, the use of LPGs seems to have supplanted the use of the more traditional RDF stores by approximately seven times: among the papers mentioning at least one GDB that were selected to appear in this review, 87% mentioned an LPG, while only 12% mentioned an RDF store, and 1% mentioned both. From the GDB technology point of view, the ones mentioned most were Neo4j—82% of the selected publications, Virtuoso—8%, and AllegroGraph—4%.

**Figure 2:**
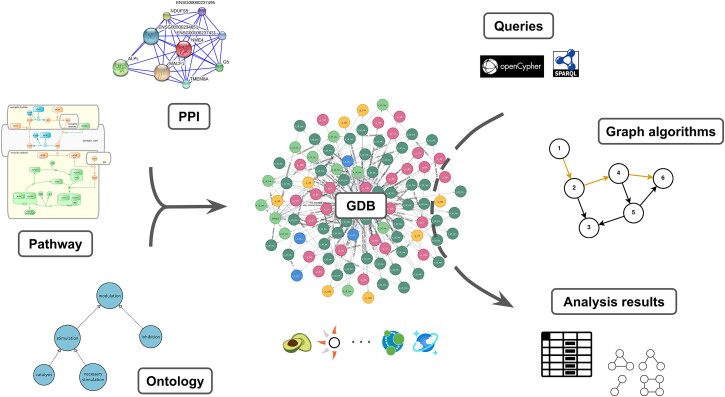
An overview of the Results section and connections between subsections: Pathway biology, ontologies, and tools, in the context of graph databases.

The following sections summarize our findings, stand-out methodologies, approaches, and resources. We bundle the results by (i) applicability in pathway biology; (ii) available ontologies, and (iii) available tools. Figure 2 shows an overview of these different sections. At the end of the Results section, we provide a use case with an example of COVID-19 resources—KGs adapted or newly developed for the COVID-19 research.

### Pathway biology

#### Process description

In systems biology, model information is mostly encoded in the Systems Biology Markup Language (SBML) [16], the Systems Biology Graphical Notation (SBGN) [17], and the Biological Pathway Exchange (BioPAX) [18]. More specifically, the SBGN Process Description language encodes biological processes [19]. It is used in pathway databases such as Reactome [20–22], PANTHER [23], Recon human metabolic network [24], and others (Table 2), in which interactions are presented in the form of molecular processes with connected regulatory proteins and complexes (see Supplementary Fig. S3 for a detailed example of an SBGN Process Description map being converted to Neo4j). Reactome is a knowledge base of biomolecular pathways not only originally stored in a relational database format but also available in the Neo4j GDB format [6, 20–22]. The Neo4j Reactome shows greatly improved query efficiency when compared to the relational database [6]. Recon2 is a genome-scale human metabolic network stored initially in SBML format with the visualization built in CellDesigner on the MINERVA platform [31]. The Neo4j version of Recon2 is available on GitHub (Table 2) for exploration and querying [26]. The StonPy tool [27] made it possible to create Neo4j resources for the Atlas of Cancer Signalling Network (ACSN) and PANTHER pathway database, with new possibilities for analysis and comparison of Reactome, PANTHER, and ACSN [28]. The StonPy library also allowed building a Neo4j instance of the COVID-19 Disease Map [27].

**Table 2:** Examples of process description-based pathway resources available in Neo4j.

Database	Content	Accessible at	Publications
Reactome	Pathways in SBML- and SBGN-compatible format	https://github.com/reactome/graph-core	[6]
Plant Reactome	Pathways in SBML- and SBGN-compatible format	https://plantreactome.gramene.org	[25]
Recon2	Metabolic pathways in SBML format	https://github.com/ibalaur/MetabolicFramework	[26]
PANTHER	Pathways built in CellDesigner in SBML- and SBGN-compatible format	Can be installed using stonpy (https://github.com/adrienrougny/stonpy)	[27, 28]
Atlas of Cancer Signalling Network	Signalling network of cancer-related mechanisms built in CellDesigner in SBML- and SBGN-compatible format	Can be installed using StonPy (https://github.com/adrienrougny/stonpy)	[27, 28]
COVID-19 Disease Map	Signalling pathways in SBML- and SBGN-compatible format focused on the COVID-19 mechanisms	https://c19dm-neo4j.lcsb.uni.lu/browser	[29]
KEGG Pathway Database	Signalling and metabolic pathways	http://biochem4j.org	[30]

The main advantage of these GDB resources is the access provided to the corresponding pathway resources, allowing their network-based exploration and analysis. These resources store the data in their own formats, but, through the Neo4j environment, the data and relationships between them can be searched, compared, and used in the same analytical pipeline [28].

#### Protein–protein interactions

Information on protein–protein interactions (PPIs) is fundamental for understanding the functioning of biological systems [32]. Well-established PPI databases are broadly available (including [33–40]).

In a graph, proteins can be represented as nodes and PPIs as edges [32, 41]. Due to their capabilities in facilitating network-based integration, querying, and analysis, GDB approaches gained popularity for managing PPI data [42]. Here, we outline the specific advantages of GDBs for the management of PPI data focusing on (i) heterogeneous data integration and exploration and (ii) support for network-based analysis and modelling. We provide several prominent examples of well-established GDBs for each group. An extended list is provided in Supplementary Table S3.

PPI networks can be extremely large and complex, involving thousands of protein/complex interactions in interconnected pathways [41]. For a proper understanding of biological systems, PPI data have to be integrated with other additional data types [32, 43]. GDBs (i) provide means for the integration of multimodal data types, such as gene expression, disease biomarkers, drug targets, pathway involvements, tissue, and cell type association [1, 44–46] and (ii) allow for flexible and expressive queries on PPI networks [47–50]. Heterogeneous data integration within one single GDB enables comprehensive analysis and interpretation of biological phenomena by considering multiple layers of systems biology simultaneously [51]. For example, the SmartGraph knowledge base integrates data on compounds and targets, focusing on drug–target interactions and PPI [52]. The Network-based Drug Repurposing and exploration (NeDRex) platform integrates several biomedical data types (including genes, proteins, drugs, and their targets) with their interrelationships and uses the inner PPI network as a central and major layer in network-based analysis aimed towards drug repurposing and disease module identification [46]. IntAct is a comprehensive open-source curated resource that provides detailed information on PPIs and molecular complexes, facilitating the exploration of interaction networks in biological systems. The IntAct Neo4j component empowers researchers to perform advanced queries and visualization of the integrated data, streamlining the computational analysis of intricate molecular networks [47]. The Protein Data Bank in Europe Knowledge Base [48–50] is a well-established open-access repository on proteomic data (3D structures, functional and biophysical annotations). A hybrid relational-GDB approach is implemented: an Oracle component that is more efficient on simple queries and a Neo4j solution that permits executing more sophisticated queries and analyses [49].

GDBs are also suitable for network-based analysis for PPI data. The graph-based algorithms implemented in GDBs (details given in the Tools section) provide means for detection of hidden patterns in interconnected data as well as for the prediction of novel associations or interactions between entities in heterogeneous biological networks involving protein interaction data [44, 46, 53, 54]. For example, SmartGraph [52] used network-based inference to perform *in silico* prediction of novel relationships between compounds and targets, exploring the complex landscape of drug–target and target–target interactions. In Mishra *et al*. [54], a combined approach of a human PPI network (integrating over 200 000 000 interactions involving >20 000 proteins) and a regulatory network was developed to explore pathologic features of neurodegeneration in amyotrophic lateral sclerosis. The Clinical Knowledge Graph (CKG) is an open-source platform that integrates data on various biomedical concepts (e.g. proteins, tissues, peptides, drugs, biological function, cellular components) and their inter-relationships from clinical experimental studies, public databases, and specialized literature. It focuses on proteomics analysis and interpretation via incorporated statistical algorithms and machine learning. The CKG uses a Neo4j GDB to manage the knowledge base composed of millions of nodes and inter-relationships and has developed a library for optimized implementation of graph-based algorithms including path finding, similarity functions, and community detection [53].

### Ontologies

An ontology is a set of concepts and relationships between these concepts that describes a domain of knowledge. Ontologies play a role in a wide variety of tasks in bioinformatics, allowing researchers to define and share a common conceptualization of a domain in a formal way. Numerous ontologies have been defined to describe different subdomains of biology and in particular systems biology [18, 55–61].

#### Ontologies and graph databases

RDF and ontologies are tightly linked technologies in the realm of the semantic web. RDF enables a linked data paradigm [62], used in ontologies to create a semantic layer that enables formal reasoning and knowledge discovery. Most ontologies available online are represented and exchanged using the Web Ontology Language (OWL) (w3.org/TR/owl-guide), which is built on top of the RDF format. Ontologies can thus be represented as RDF triples and queried using SPARQL (w3.org/TR/rdf-sparql-query). Some RDF stores also include reasoning capabilities supporting direct OWL-based inferences (e.g. AllegroGraph and Virtuoso). Most of the mentioned systems biology ontologies are stored using RDF stores, but some also use Neo4j as their endpoint (Table 3, see Supplementary Table S4 for an extended list). Tools such as Owl2Neo4j [68] may be used to store an OWL ontology in a Neo4j database automatically.

**Table 3:** Examples of systems biology ontologies that are stored in GDBs.

Ontology	Content	GDB	OWL	Accessible at	Publications
Disease Ontology	Medical terms and human diseases	Neo4j	Yes	https://disease-ontology.org	[59]
Knowledge Base of Biomedicine	Biomedical data	AllegroGraph or Virtuoso	Partially	Installed locally via https://github.com/drlivingston/kabob	[63]
Protein Ontology	Taxon-specific and taxon-neutral protein-related entities	Virtuoso	Yes	https://proconsortium.org	[64, 65]
Human Phenotype Ontology	Phenotypic abnormalities in humans	Unknown but part of the Monarch Initiative (https://monarchinitiative.org) that uses RDF and Neo4j	Yes	https://hpo.jax.org/app	[66, 67]
Unified Phenotype Ontology	Organism-specific phenotypes	Unknown but part of the Monarch Initiative (https://monarchinitiative.org) that uses RDF and Neo4j	Yes	https://ols.monarchinitiative.org/ontologies/upheno2	[66]

#### Ontologies for data integration in graph databases

Ontologies may be used as backbones to integrate data from different sources into one database. In the context of GDBs, this may be facilitated by the tight integration of ontologies into the RDF framework. The (semi-)automatic integration process generally relies on the transformation of heterogeneous data into uniform ontology-backed RDF triples using rules (e.g. the Knowledge Base of Biomedicine (KaBOB) [63]), probabilistic models (e.g. GORouter [69]), or shared guidelines (e.g. Bio2RDF [70]). The integration process may result in unique RDF stores (KaBOB, GORouter) or a series of individual although homogeneous stores that can be queried using federated SPARQL queries (Bio2RDF) [71].

#### Ontology-based graph database queries

Data can be retrieved from GDBs using database-specific query languages. While all RDF stores may be queried using SPARQL, there is no unique standard language for LPG databases (see Table 1). A means to overcome this heterogeneity in query languages is to build systems that allow users to query databases in natural language. In some systems, the transformation process is knowledge-based and guided by the ontology that backs the GDB [72]. For example, the OntoNLQA framework can be used to automatically answer natural language questions based on parasite immunology data stored in an RDF store backed by an ontology [73]. Ontologies may also be used to check the correctness of user input queries in the context of GDBs [74].

### Tools

Graph algorithms play an important role in data science and systems biology in particular, as they can be integrated into frameworks for analysing and extracting insights from highly interconnected datasets, providing a better understanding of the underlying data. They can be used to explore existing relationships and predict new connections across metabolic, signalling, and regulatory networks and create visually appealing representations of biomedical networks, facilitating the exploration and interpretation of complex datasets. The following are some examples of common analytical approaches for biological data using graph algorithms. An extended list of tools is given in Supplementary Table S5.

Pathfinding aims to identify the shortest path between two entities, making it useful in exploring the biological context [7, 75]. For example, the Neo4j-based resource GREG combines five types of regulatory processed data (transcription factors, regulatory noncoding RNAs, chromatin interactions, protein complexes, and cofactors). Using graph traversal algorithms, it is possible to determine if two nodes are directly connected or if their relationship is mediated by other nodes in the integrative network. This helps determine the shortest path between a transcription factor and its target gene or between a noncoding RNA and its associated genomic region, facilitating the exploration of regulatory pathways involved in gene expression and regulation. Identifying a short path may suggest direct regulation, while longer paths involving multiple intermediate molecules indicate more complex regulatory networks. A potential connection could suggest new biological mechanisms [76].

Connectivity analysis allows exploration of the neighbourhood of a node of interest, revealing the strength of functional and structural links between biological entities and ‘centerpoints’ for different regions of the graph. It further serves to analyse the flow of information inside the network and to explore similarities between different entities based on their common connections and properties. Neo4j-based Graffinity is an example of a connectivity analysis tool [77], applied to a connectome (a graph of connections between cells) in the retina. The authors detected a previously unknown anomalous pathway between cone cells and rod cells, finding an intermediate node in the pathway with unexpected connections to cone cells. Pinpointing the specific synapses responsible for this anomaly, the authors discovered that it was an annotation error. Despite previous analysis of this connectome at a broader level of detail, fine-scale annotation errors remained, and they were revealed when conducting visual connectivity analysis.

Subgraph mining identifies frequently occurring patterns (subgraphs) in complex graph structures [78]. In systems biology, subgraph mining is used to identify important molecular interactions and biological pathways in large-scale biological data such as PPI networks or metabolic pathways and to identify coding patterns and overlap of systems biology models [79].

Visual exploration allows us to see the relationships within the data and perform visual network analysis. For example, starPepDB supports visual exploration of integrated bioactive peptide data gathered from a large array of databases [80]. Also, web-based user-friendly applications that integrate a GDB component facilitate bioinformatics data extraction, visualization, and analysis. One such application is BioGraph, which uses a collection of heterogeneous data from a variety of bioinformatics resources. An important analytical feature is its own query language called Gremlin, as it supports both declarative and imperative queries. This allows for an explicit implementation of the traversal algorithms that a query will utilize, offering advanced and complex custom graph-based algorithms [81].

### Systems biology use-case: COVID-19 resources

During the COVID-19 pandemic, a scientific effort of unprecedented global scale has been made, resulting in a significant number of resources and community projects using GDBs to integrate and explore the rapidly emerging new data about SARS-CoV-2 infection and COVID-19 disease [47, 82–91] (see Supplementary Table S6 for a list of GDB-based COVID resources). For example, GDB approaches have contributed to the development of (i) molecular pathways [47], (ii) clinical trials and drug repurposing [82–85], (iii) ontology resources related to COVID-19 [86–88], and (iv) application of graph-based methods for the exploration of COVID-19 mechanisms, comorbidities, and risk factors [88–90]. A classification of the COVID-19 KGs using GDBs based on their main application domain is provided in Chatterjee *et al*. [91]. Here, we discuss selected GDBs for COVID-19 data focusing on pathway biology, resources developed using ontologies, and tools used in the COVID-19 research.

A particular class of GDB resources focused on integrating heterogeneous COVID-19 data to facilitate data exploration and visualization of molecular pathways and disease mechanisms [47, 84, 86, 87]. For example, the IntAct Coronavirus interactome dataset integrates PPIs and RNA–protein interactions involving SARS-CoV-2 and SARS-CoV and can be explored in the Neo4j version of IntAct [47]. KG-COVID-19 (Neo4j-based) [84] and COVID-19 KG (Virtuoso-based) [86] are comprehensive knowledge bases for machine learning applications and downstream analysis in COVID-19 drug repurposing. KG-COVID-19 integrates primarily data on drug targets, protein interactions, protein functional annotations, and disease ontologies [84]. The COVID-19 Knowledge Graph is developed using text mining and relevant curated biological databases [86]. Data exploration and visualization of KGs are also employed in several comprehensive COVID-19 community projects, including HealthEcco (healthecco.org) [87] and COVID-19-Net (github.com/covid-19-net/covid-19-community). HealthEcco integrates COVID-related data such as publications and patents, clinical trial data, biomedical data, and computational systems biology models into a Neo4j GDB to provide a single point of access to these diverse data sources. The COVID-19-Net project uses a Neo4j approach to integrate heterogeneous biological data types (both health- and pathogen-related) with environmental characteristics to facilitate the exploration of COVID-19 mechanisms by looking at interdependencies among host–pathogen–environment systems.

COVID-19 GDBs are backed up by ontologies to facilitate semantic integration of data from multiple sources. Semantic relationships are enriched by integrating knowledge from several public biomedical repositories and ontologies [34, 66, 67, 92–95]. The KGEV framework uses Neo4j to store and query the data and can be extended to other diseases. The gcCov is a coronavirus genotype–phenotype KG based on a semantic web framework (employing RDF and Neo4j) and open linked data. This database provides a resource for structural and sequence similarities among coronaviruses and may therefore aid in the identification of cross-neutralizing antibodies that bind to multiple CoV antigens, which may be relevant for the treatment of SARS-CoV-2 infections [90].

COVID-19 GDBs have been also used to explore candidates for drug repurposing using computational modelling approaches [82–85]. For example, a novel method using neural networks (involving several graph completion algorithms) and literature curation approaches was developed for the identification of candidates for COVID-19 drug repurposing. The work uses Neo4j to store semantic relationships among the data (e.g. relationships on inhibition, interaction, association, causality between drugs, and other biological concepts) and to help with navigation and visualization of the integrated resources. The Neo4j functionality was also used in a computational analytical step to evaluate the plausibility of several highly ranked drug candidates returned by the graph-based completion component [82]. Identification of possible drugs for treatment can also be achieved by a graph neighbourhood search, as performed on a COVID-19 KG constructed using the KGEV framework [88]. In addition, a shortest-path approach identified similarities in pathways (alterations) in obese people and COVID-19 patients. In COVID-19 pharmacology research, a workflow for semiautomated integration of multimodal data was used to develop the Neo4COVID19 resource, which describes a network of host–host, host–pathogen, and drug–target interactions for COVID-19 [85].

## Discussion

### Challenges and lessons learned

#### Training and documentation for graph databases help to use them efficiently

GDBs, and in particular LPGs, are a relatively new technology compared to relational databases. An effort to use these tools efficiently is ongoing, and new techniques are continuously developed.

Knowing the data model and being familiar with the query language are key steps for efficient use of GDBs. The LPG ecosystem is not completely mature and still undergoes rapid changes. LGPs notably lack a standardized query language (such as SQL for relational databases or SPARQL for the RDF), despite progress on openCypher (opencypher.org) and International Organization for Standardization Graph Query Language (gqlstandards.org). Therefore, it is of particular importance that the developed resources and software are well documented and that query examples are provided and explained by initial developers.

#### Integrated resources and sustainability

The term ‘integrated resources’ refers to GDBs that assimilate data from multiple sources. Integrated resources facilitate (i) discovery of new connections across data from multiple sources (e.g. pathway biology) and (ii) semantic enrichment by combining data and ontologies. They offer a single query language and access to multiple databases via a single platform.

A large portion of the reviewed GDBs for systems biology are integrated resources (see Supplementary Table S7 for a list of primary resources and Supplementary Table S8 for a list of integrated resources), which suggests that relational databases are still the main technology for primary data sources. This could be explained by the fact that (i) GDBs are still a new technology compared to relational databases, (ii) they might be difficult to adopt, and (iii) they are less efficient than relational databases for some types of queries (e.g. complex queries with aggregates) or for structured data that are not densely interconnected [96].

GDBs are adequate for data integration tasks: they are schema-optional, they are efficient for visualizing and retrieving highly connected data, and they are compatible with ontologies. However, GDBs still face challenges inherent to the integration of heterogeneous data types originating from multiple resources and the sustainability of these integrated resources [97–99]. This latter issue is particularly significant, as among the 93 publications that report accessible resources for data integration, only 20 are regularly updated (see Supplementary Table S8). These difficulties can be addressed with standardized approaches (see Efforts toward a Uniform Development of Knowledge Bases) or with the use of specific GDB technologies, such as federated queries [100].

### Perspectives

GDBs are suitable for systems biology and will support future automated model generation and machine learning tasks. However, they need to be standardized, documented, and maintained to unlock their full potential. Therefore, key points when planning a GDB application are (i) building on established approaches that aim at standardizing KG creation, (ii) following the principles of Findability, Accessibility, Interoperability and Reusability (FAIR) [101] for the data included and the principles of Transparency, Responsibility, User focus, Sustainability and Technology (TRUST) [102] for the KG itself, and (iii) automating the GDB maintenance.

#### Pathway resources available in process-description-type and activity-flow-type formats

We anticipate that in the future more pathway resources will be made available in GDB environments, ideally using standard compatible formats such as the Systems Biology Graphical Notation (SBGN) [28, 103]. For example, the OmniPath resource [104] is a collection of databases, including a signalling network database and a database on posttranslational modification of enzymes. Information is integrated from >100 resources (omnipathdb.org/info). The content representation is compatible with the SBGN Activity Flow standard language [105] and can be accessed via Python and a Cytoscape plug-in [106]. The Pathway Commons [107, 108] integrates pathway information from 22 databases (pathwaycommons.org). Its content is represented using mainly the BioPAX language [18] with visualization available in SBGN [17]. This extensive resource covers 2.3 million interactions [107], accessible via Java, R, Python and Javascript. Both SBGN Process Description and Activity Flow conceptual types of relationships are included. A GDB instance would facilitate network-based exploration and analysis of the pathway content.

#### Elasticsearch and graph databases

Elasticsearch (elastic.co) is a distributed open-source search and analysis platform that can process large-scale data of various types, including text, numerical, structured, and unstructured data. Elasticsearch is based on indexing, where an index is a collection of documents related to each other. It uses a data structure called an inverted index that connects every unique word appearing in any document to all the documents of the collection it appears in, allowing fast full-text searches. When presented with a new document, Elasticsearch stores it and re-builds an inverted index.

Elasticsearch and GDB technologies have been recently combined, for example, creating optimized systems for semantic indexing and classification of biomedical literature [109] or knowledge bases that enable the exploration of drug molecular mechanisms for precision medicine [110]. In systems biology, the Alliance of Genome Resources, which integrates data from the major model organisms databases, uses Neo4j as a database and the Elasticsearch technology as a search service [111]. To this end, the Alliance harmonized data models of the different sources and curation workflows. As a result, all sources can be integrated into a single database with a unified data model, which facilitates queries spanning over several organisms and enables cross-organism investigation.

#### Efforts towards a uniform development of knowledge bases

Several challenges arise with the rapidly increasing number of GDBs in the field of systems biology and systems medicine [44, 45, 53, 112]. One of the challenges faced by GDBs is redundancy. If sources without standardized metadata schema are connected to each other, duplicate nodes and relations are introduced. Identification and removal of such duplicates is time-consuming and may require manual intervention. Additionally, the design of a high-quality and well-maintainable GDB requires informed decisions about the specific GDB approach, the appropriate data model, the relevant semantic enrichment, etc. For many researchers, specifically in the applied biological and clinical domains, such decisions do not lie in their field of expertise, easily resulting in shortcomings of the designed GDBs. To overcome the described problems and to improve the quality of the resulting GDBs, the systems biology community started to design methods and implement tools that harmonize and standardize GDB development.

Within the Biomedical Data Translator project [113], the so-called Knowledge Beacons API allows accessing knowledge sources and discovering shared semantics [114]. This work provided access to several important GDB resources, such as SemMedDB, HMDB, or Biolink, but required labour-intensive specific indexing and query definitions for each resource. Later, RTX-KG2 [115] was developed to integrate biomedical concepts and their relationships from 70 different knowledge sources, including ChEMBL [116], DrugBank [117], KEGG [118], Reactome [20] and UniProtKB [94]. To deal with this unprecedented amount of data sources, it was necessary to standardize the schema and semantic layers. The resulting GDB conforms to the Biolink model [60] and includes provenance information to maximize interoperability.

BioCypher (biocypher.org) is a framework for the development of integrated biology-related GDBs [119], freely available (github.com/biocpher/biocypher) and reusable under the MIT licence. BioCypher facilitates the integration of diverse sources into one Neo4j GDB. It uses a modular approach based on project-specific input and output adapters and relies on the Biolink data model [60] for structuring the integrated information. The available and reusable BioCypher adapters are represented as a meta-graph (github.com/biocpher/meta-graph) based on the Biomedical Resource Ontology (BRO) [120] (github.com/biocpher/biomedical-resource-ontology). In summary, the BioCypher framework makes the implementation of a GDB accessible to researchers with limited technological knowledge and it facilitates integration and harmonization of diverse data sources. Several well-established systems biology resources have already joined this project, such as the CKG [53], the OTAR KG [121], or the HealthEcco project (healthecco.org) [87].

## Conclusion

We observe a rapid increase in the use of graph databases (GDB): while in 2012, only 17 PubMed publications cited any of the GDB approaches mentioned in this review, there were >190 in 2022. In systems biology, GDBs have been proven efficient for storing data that are naturally organized in the form of graphs, such as pathways and molecular networks. For this type of data, where exploration comes to follow nodes along paths, GDBs turn out to be more efficient than relational databases, since they are less computationally expensive. The GDB approach also offers significant additional advantages (schema-optional, better visualization, embedded graph algorithms) that all together make it a great candidate for data integration, exploration and analysis in systems biology. We observe a growing number of publicly available GDB-based KGs that integrate data from multiple sources and often constitute substantial knowledge bases on more generic (e.g. human cancer) or more focused topics (e.g. COVID-19) of systems biology. The construction of such KGs often relies on nonsustainable workflows that fetch and merge data from the desired sources into one GDB, sometimes backed by ontologies that help structure the used data model. While these KGs offer readily and efficiently accessible data on specific systems biology topics, the way they are built and their growing number bring consequential issues, such as their redundancy, heterogeneity, and sustainability. These issues may be solved in the future by applying standardized common workflows and data models for building KGs and by organizing their construction and maintenance around durable communities or consortia.

## Methods

In this systematic review, we performed the following steps (see Supplementary Fig. S4 for a graphical summary).

- First, we analysed the use of GDB technologies as reported in the DB-Engines (db-engines.com, reference date Sep 2023) and prepared a list of the top 16 GDBs (Table 1).
- We then proceeded with the automatic retrieval of publications from PubMed and PMC that use the term ‘graph database’ or mention specific GDBs such as AllegroGraph, ArangoDB, GraphDB, and Neo4j (Table 1). The specific queries can be found in Supplementary Methods.
- Next, publications were manually reviewed by two reviewers and shortlisted using the following criteria: the use of a specific GDB technology and the applicability in systems biology. For integrated resources, we had an extra criterion on their sustainability, which assessed whether the resource was available to be queried or at least the source code was made publicly available. Publications were grouped into the following categories: reviews, methods, software, primary resources, integrated resources, and ontologies.
- Finally, the shortlisted publications are further refined and discussed in the text of this review, focusing on the following major topics: pathway biology, ontologies, tools, and applications for COVID-19 research. Priority for selection was given to the projects that are actively maintained and are potentially likely to be reused.

More details on methods and queries used are provided in Supplementary Methods. The complete annotated list of publications with corresponding PubMed and DOI identifiers is provided in Supplementary Table S9. Updated versions of this list will be available at github.com/ilyamazein/gdbreview/tree/main/annotated.

**Key Points**

- Graph databases (GDBs), which provide a natural fit for network-based representation of biological information, are becoming increasingly popular as a way to manage and query heterogeneous data and to provide new insights into data connections.
- Knowledge graphs facilitate the discovery of unexpected relationships across integrated multimodal data that can lead to the generation of new hypotheses in systems biology.
- This review is based on 681 systematically identified GDB-related publications from the fields of biology and bioinformatics in PubMed and PubMed Central repositories, further filtered down to 179 publications based on applicability in systems biology.
- We outline the prospects of applying GDBs in systems biology with technologies such as Elasticsearch.
- We highlight the ongoing efforts towards the development of unified GDB platforms for integration and exchange of heterogeneous biomedical data between multiple projects.

## Supplementary Material

Supplementary_bbae561

Table_S9_bbae561

## Data Availability

All relevant data are provided within this publication and supplementary files. An updated version of the annotated tables with all relevant publications is available via github.com/ilyamazein/gdbreview.
