# Supplementary material for: Graph databases in systems biology: a systematic review
Source: Brief Bioinform. 2024 Nov 20;25(6):bbae561. doi: 10.1093/bib/bbae561 (PMC11578065; doi:10.1093/bib/bbae561)
Supplement: Supplementary_bbae561 [file supplementary_bbae561.docx]

Supplementary material

*Graph databases in systems biology: a systematic review* by Mazein *et al.*

[**Supplementary figures 1**](#_xysi4f62mlb)

[Figure S1: Two types of graph models: RDF and LPG 1](#_ijfd7xjkz5jp)

[Figure S2: Number of publications mentioning a graph database 3](#_ywmawmjzh0ks)

[Figure S3: Pathway conversion from SBGN to Neo4j 4](#_d5edic5ck56i)

[Figure S4: Graphical summary of the publication workflow 4](#_byldne6cdcuu)

[**Supplementary tables 4**](#_myiwuxdj8tnh)

[Table S1: Number of selected publications and total number of publications per category 4](#_d7dirmrxgplq)

[Table S2: Number of selected publications per section of the review 5](#_b5tervu9jouv)

[Table S3: List of systems biology related PPI resources available in the form of a GDB 5](#_iul37arxcy3r)

[Table S4: List of systems biology related ontologies available in the form of a GDB 9](#_fzkcmct13n02)

[Table S5: List of systems biology related tools using a GDB 14](#_vdcxsbwl426u)

[Table S6: List of COVID related resources available in the form of a GDB 26](#_yk14mpf9pasr)

[Table S7: List of systems biology primary resources available in the form of a GDB 28](#_r3rfk5zevjxt)

[Table S8: List of systems biology integrated resources available in the form of a GDB 29](#_1gdt6skkclk5)

[**Supplementary methods 48**](#_ja3x7nivlkdf)

[PubMed and PMC search queries 49](#_1fob9te)

[Python script to create the results table 50](#_3znysh7)

[Manual review: criteria for inclusion/exclusion and subdivision into categories 50](#_2et92p0)

# Supplementary figures

## Figure S1: Two types of graph models: RDF and LPG


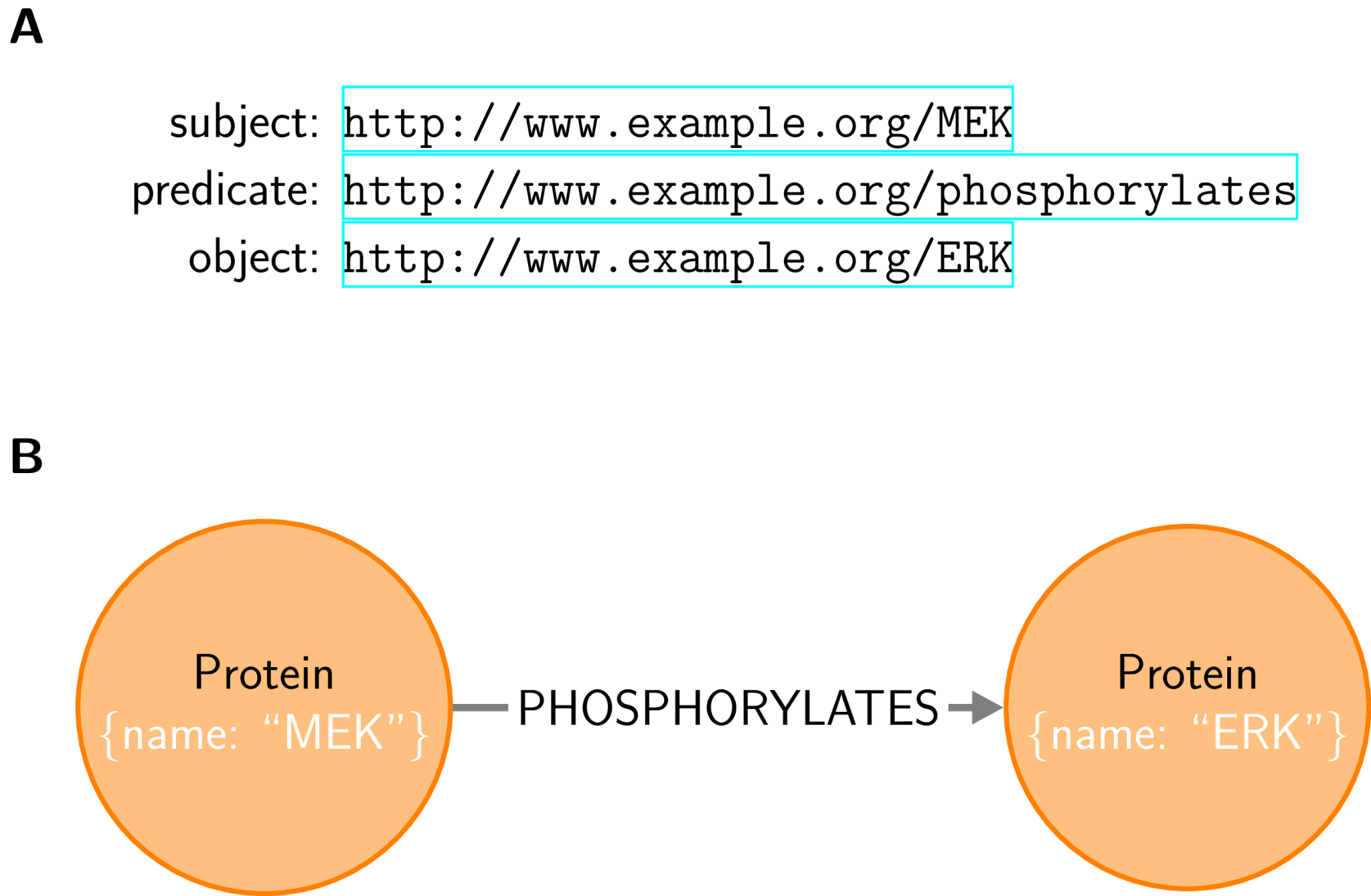


**Figure S1:** Two types of graph models: RDF and LPG. This example shows how the phosphorylation of extracellular signal-regulated kinase (ERK) by mitogen-activated protein kinase kinase (MEK) can be modeled under the two types of graph models. This process is a single step of a phosphorylation cascade involved in many different signaling pathways, including G protein coupled receptor-activated pathways and cell cycle regulation. **A.** An example of an RDF triple stating “MEK phosphorylates ERK”. The triple is composed of a subject (“MEK”), a predicate (“phosphorylates”), and an object (“ERK”), each identified using an IRI (in this case a URL). **B.** An LPG example, representing the same information as in A. Nodes are represented with circles. Both nodes are labeled as “Protein”, and have one property with the key “name”. The relationship in the graph is represented with an edge, where the type is “PHOSPHORYLATES”. In this example, the relationship (edge) does not have a property, but if needed, it can store additional information such as cardinality, cell type, etc.

## Figure S2: Number of publications mentioning a graph database


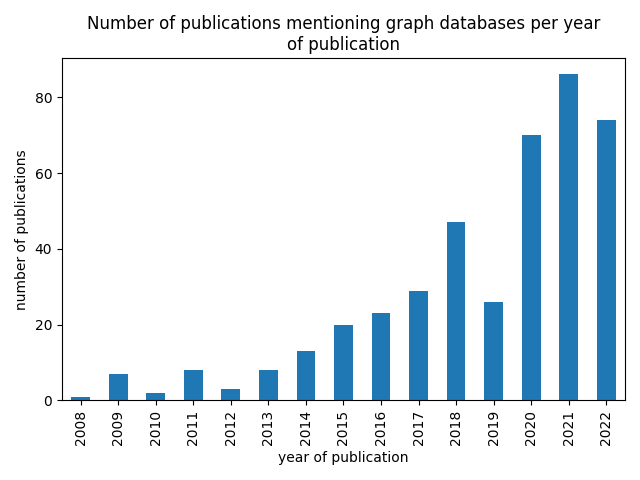
 **Figure S2:** number of publications mentioning a graph database (as retrieved by our queries, see Methods for more details) per year of publication. The number of publications for 2023 is not included since our cut-off date is 31/03/2023.

## Figure S3: Pathway conversion from SBGN to Neo4j


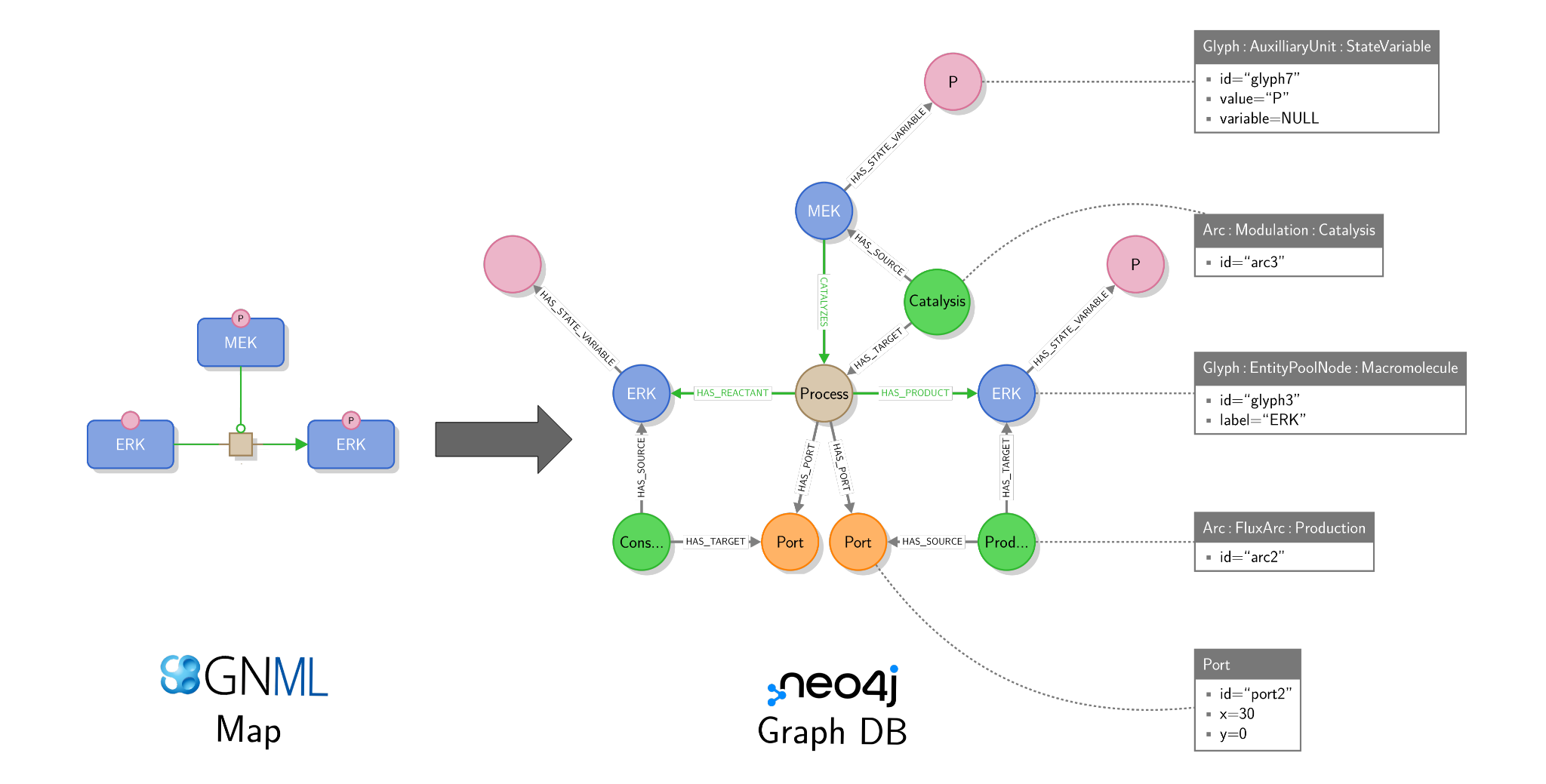


**Figure S3:** graphical example of an SBGN Process Description map being converted into a graph database format in Neo4j (adapted from the StonPy publication, listed in Table S5 - DOI: 10.1093/bioinformatics/btad100).

## Figure S4: Graphical summary of the publication workflow


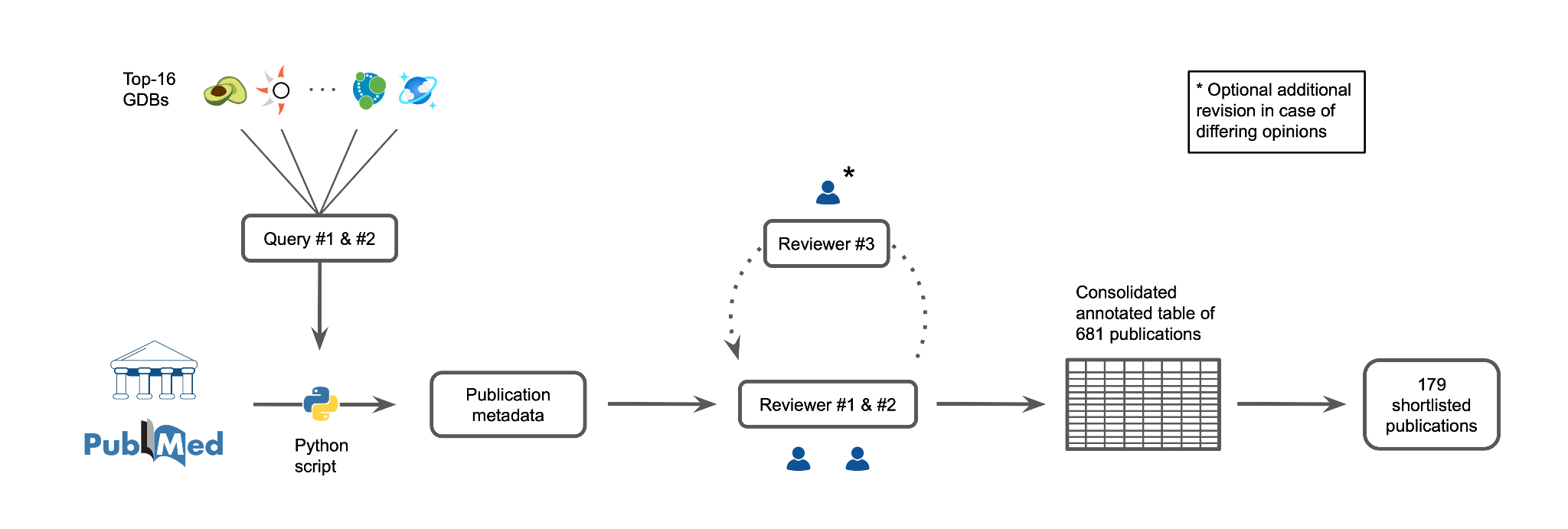


**Figure S4:** graphical summary of the steps in the shortlisting workflow.

# Supplementary tables

## Table S1: Number of selected publications and total number of publications per category

| **Category** | **Number of selected publications (total number of publications)** |
| --- | --- |
| REVIEW | 14 (64) |
| METHOD | 28 (238) |
| SOFTWARE | 57 (163) |
| PRIMARY | 3 (6) |
| INTEGRATED | 93 (206) |
| ONTOLOGY | 21 (45) |
| OTHERS | 3 (103) |

## Table S2: Number of selected publications per section of the review

| **Section** | **Subsection** | **Number of selected publications** |
| --- | --- | --- |
| Pathway biology | Process Description | 7 |
|  | PPI | 19 |
| Ontology |  | 21 |
| Tools |  | 85 |
| COVID |  | 13 |

##

## Table S3: List of systems biology related PPI resources available in the form of a GDB

| **Resource** | **Content** | **Accessible at** | **GDB** | **Publication** |
| --- | --- | --- | --- | --- |
| PDBe Aggregated API | A KG that integrates the content of the well-established Protein Data Bank resource | gitlab.ebi.ac.uk/pdbe-kb/services/pdbe-graph-api; pdbe.org/graph-api | Neo4j | 10.1093/bioinformatics/btab424 |
| SmartGraph | A network pharmacology platform | [smartgraph.ncats.io/](https://smartgraph.ncats.io/) | Neo4j | 10.1186/s13321-020-0409-9 |
| Hetionet (Project Rephetio) | A resource integrated millions of biomedical studies | [neo4j.het.io](http://neo4j.het.io) | Neo4j | 10.7554/eLife.26726 |
| GeneDiseaseRepositioning | An integrated Data Driven Approach to Drug Repositioning Using Gene-Disease Associations | [bitbucket.org/ncl-intbio/genediseaserepositioning/src/master/](https://bitbucket.org/ncl-intbio/genediseaserepositioning/src/master/) | Neo4j | 10.1371/journal.pone.0155811 |
| Reactome GDB | The GDB (neo4j) version of the Reactome KB | [reactome.org](https://reactome.org) | Neo4j | 10.1093/nar/gkx1132 |
| OTAR interactome | Integrated resource + graph-based analytics | ftp://ftp.ebi.ac.uk/pub/databases/intact/various/ot_graphdb/current) | Neo4j | 10.1038/s41588-023-01327-9 |
| PDBe-KB | describes the GDB components for the Protein Data Bank in Europe | [www.ebi.ac.uk/pdbe/pdbe-kb/](https://www.ebi.ac.uk/pdbe/pdbe-kb/) | Neo4j | 10.1002/pro.4439 |
| NeDRexDB | Describes an integrated framework that supports network-based analytics for disease module identification and drug repurposing | [neo4j.nedrex.net/](http://neo4j.nedrex.net/) | Neo4j | 10.1038/s41467-021-27138-2 |
| SEARCHIN | An analytical pipeline that integrates several existent functionalities and was developed to prioritize interactions between ligands and receptors in amyotrophic lateral sclerosis | [github.com/Califano-lab/SEARCHIN](https://github.com/Califano-lab/SEARCHIN) | Neo4j | 10.1038/s41467-020-19177-y |
| Tabloid Proteome | Integrates content on inferred protein-protein association | [iomics.ugent.be/tabloidproteome](http://iomics.ugent.be/tabloidproteome) | Neo4j | 10.1093/nar/gkx930 |
| NetworkAnalyst | A network-based framrwork for integration, analysis and visualisation of gene expression profiling | [www.networkanalyst.ca/](https://www.networkanalyst.ca/) | Neo4j | 10.1093/nar/gku443 |
| IntAct | IntAct GDB; molecular interaction data from the IntAct resource | [www.ebi.ac.uk/intact/download](https://www.ebi.ac.uk/intact/download); github.com/intact-portal | Neo4j | 10.1093/nar/gkab1006 |
| PDBe-KB | Structural information; annotations derived from or relevant to the Protein Data Bank (PDB); Protein Data Bank in Europe – Knowledge Base | pdbe-kb.org; pdbe-kb.org/graph-download; pdbe-kb.org/graph-download | Neo4j | 10.1093/nar/gkab988 |
| Inxight Drugs; NCATS Inxight Drugs | An extension of the G-SRS codebase; Drug database: 125 036 product ingredients, including 2566 US approved drugs, 6242 marketed drugs, and 9684 investigational drugs | drugs.ncats.io; drugs.ncats.io/ | Neo4j | 10.1093/nar/gkab918 |
| GraphOmics | The GraphOmics is a user-friendly KG to explore and integrate multiple omics datasets (transcriptomics, proteomics and metabolomics) and support hypothesis generation. Integration on top of Reactome's GDB | [graphomics.glasgowcompbio.org/](https://graphomics.glasgowcompbio.org/) | Neo4j | 10.1186/s12859-021-04500-1 |
| RPGeNet v2.0 | A tool to visualize the interactome network of visual disorder genes | [compgen.bio.ub.edu/RPGeNet](https://compgen.bio.ub.edu/RPGeNet) | Neo4j | 10.1093/database/baz120 |
| ROBOKOP | A tool for KGs exploration | [robokop.renci.org/](http://robokop.renci.org/) | Neo4j | 10.1093/bioinformatics/btz604 |
| SPOKE | Knowledge graph for precision medicine built from integrating 41 databases and based on ontologies | [spoke.rbvi.ucsf.edu/](https://spoke.rbvi.ucsf.edu/) | Neo4j | 10.1093/bioinformatics/btad080 |
| Fragment Graph DataBase (FGDB) | A graph database of ligand fragments from the Protein Data Bank | [biochimica3.bio.uniroma3.it/fragments-web/](http://biochimica3.bio.uniroma3.it/fragments-web/) | Neo4j | 10.1093/database/baac044 |

## **Table S**4: List of systems biology related ontologies available in the form of a GDB

| **Resource** | **Content** | **Accessible at** | **GDB** | **Publication** |
| --- | --- | --- | --- | --- |
| NG-Tax 2.0 | FAIR high-throughput analysis and classification of marker gene amplicon sequences | [wurssb.gitlab.io/ngtax](http://wurssb.gitlab.io/ngtax) | RDF | 10.3389/fgene.2019.01366 |
| BioKNO | Biological Knowledge Network Ontology (BioKNO), a lightweight ontology for the represention of biological-related knowledge networks | [github.com/Rothamsted/bioknet-onto](https://github.com/Rothamsted/bioknet-onto) | Neo4j | 10.1515/jib-2018-0023 |
| BKO | The Bricks Ontology (BKO), an ontology describing standardized representations of recurring concepts in systems biology | [www.sbgnbricks.org](http://www.sbgnbricks.org) | Neo4j | 10.1093/bib/bbab049 |
| SciGraph | A presentation of the Monarch platform, which offers access to integrated biological data across different species | [github.com/SciGraph/SciGraph](https://github.com/SciGraph/SciGraph) | Neo4j | 10.1093/nar/gkz997 |
| KaBOB | 18 databases; Knowledge Base Of Biomedicine | [github.com/drlivingston/kabob](https://github.com/drlivingston/kabob) | AllegroGraph; Virtuoso (RDF) | 10.1186/s12859-015-0559-3 |
| Bio2RDF | An integrated resource for various types of biological data, that enables ontology-based queries | [bio2rdf.org/](https://bio2rdf.org/) | Virtuoso (RDF) | 10.1186/2041-1480-4-S1-S1 |
| Protein Ontology Open Linked Data | Protein Ontology (PRO) Linked Open Data SPARQL endpoint (resource) and GUI/Restful APIs to access it (Tool). This resource links the PRO to other resources. | [lod.proconsortium.org/](https://lod.proconsortium.org/) | Virtuoso (RDF) | 10.1038/s41597-020-00679-9 |
| Drug Target Ontology | The Drug Target Ontology: formalized and standardized classifications and annotations of druggable protein targets | [drugtargetontology.org](http://drugtargetontology.org) | Neo4j | 10.1186/s13326-017-0161-x |
| BioTCM-SE | An instantiated ontology for the integration of modern biology and traditional Chinese medicine concepts and data, and a query platform for this ontology | [www.biotcm.org/biotcm-se/indexpage/index.html](http://www.biotcm.org/biotcm-se/indexpage/index.html) | Virtuoso (RDF) | 10.1155/2014/957231 |
| NeXO | A gene ontology built computationally from gene networks backed by a database and visualization platform | [www.nexontology.org/](http://www.nexontology.org/) | Neo4j | 10.1093/nar/gkt1192 |
| GORouter | An RDF model which encodes heterogeneous original data in a uniform RDF format, creates additional ontology mappings between GO terms, and introduces a set of inference rulebases | [www.scbit.org/gorouter/](http://www.scbit.org/gorouter/) | Oracle 10g NDM (RDF) | 10.1186/1471-2105-9-S1-S6 |
| neo4j2owl | pipeline to develop cell type ontology for brain tissues | [github.com/VirtualFlyBrain/neo4j2owl](https://github.com/VirtualFlyBrain/neo4j2owl) | Neo4j | 10.1038/s41597-022-01886-2 |
| CORAL | it discusses a framework for integrating and analyzing complex biological data. | [github.com/jmchandonia/CORAL](https://github.com/jmchandonia/CORAL) | ArangoDB | 10.1093/gigascience/giac089 |
| Monarch Knowledge Graph | it is a resource for ontologies and ontology-encoded knowledge | [github.com/SciGraph/SciGraph/](https://github.com/SciGraph/SciGraph/) | Neo4j | 10.1093/nar/gkw1128 |
| Protein onology | the RDF-based system for the Protein Ontology | [proconsortium.org/](https://proconsortium.org/) | Virtuoso (RDF) | 10.1093/nar/gkw1075 |
| OntoFox | integrative resource for ontologies | [ontofox.hegroup.org/](http://ontofox.hegroup.org/) | Virtuoso (RDF) | 10.1186/1756-0500-3-175 |
| WebProv | This is a neo4j-based system for semantic connection among systems biology models; Relating simulation studies by provenance—Developing a family of Wnt signaling models | [github.com/SFB-ELAINE/WebProv](https://github.com/SFB-ELAINE/WebProv) | Neo4j | 10.1371/journal.pcbi.1009227 |
| DO | A comprehensive knowledge base of 8043 inherited, developmental and acquired human diseases | [disease-ontology.org/](http://disease-ontology.org/) | Neo4j | 10.1093/nar/gkr972 |
| SPOKE | Knowledge graph for precision medicine built from integrating 41 databases and based on ontologies | [spoke.rbvi.ucsf.edu/](https://spoke.rbvi.ucsf.edu/) | Neo4j | 10.1093/bioinformatics/btad080 |
| BioLink | The BioLink model is a standardised data model for biomedical KGs to support integration and interoperability of these KGs | [github.com/biolink/biolink-model](https://github.com/biolink/biolink-model) | Neo4j | 10.1111/cts.13302 |
| Neuron Phenotype Ontology (NPO) | A standardized and automatable approach for naming cell types and normalizing their constituent phenotypes using identifiers from community ontologies as a common language | [bioportal.bioontology.org/ontologies/NPOKB](https://bioportal.bioontology.org/ontologies/NPOKB) | OWL | 10.1007/s12021-022-09566-7 |
| PDX Finder | A comprehensive open global catalog of patient-derived tumor xenograft models and their associated datasets | [www.pdxfinder.org](http://www.pdxfinder.org) | Neo4j | 10.1093/nar/gky984 |
| SATORI | An integrative search and visual exploration interface for the exploration of biomedical data repositories | [satori.refinery-platform.org/](http://satori.refinery-platform.org/) | Neo4j | 10.1093/bioinformatics/btx739 |
| ChemRDF | A database system that adresses certain shortcomings of RDF databases that make their use restrictive or challenging for common users, demonstrated on the ChEBI database | [bioinfo.uochb.cas.cz/projects/chemRDF](https://bioinfo.uochb.cas.cz/projects/chemRDF) | Virtuoso (RDF) | 10.1186/s13321-016-0144-4 |
| OBIB | Merging two ontologies, developed on the basis of Open Biological and Biomedical Ontologies Foundry, using a modular approach, creating OBIB - a novel ontology for biobanking, that runs on Stardog. | [purl.obolibrary.org/obo/obib.owl](http://purl.obolibrary.org/obo/obib.owl) | Stardog (RDF) | 10.1186/s13326-016-0068-y |

## **Table S**5: List of systems biology related tools using a GDB

| **Software** | **Content** | **Software page** | **GDB** | **Publication** |
| --- | --- | --- | --- | --- |
| Pheno4J | annotated genetic variants and well phenotyped patients | [github.com/phenopolis/pheno4j](https://github.com/phenopolis/pheno4j) | Neo4j | 10.1093/bioinformatics/btx397 |
| PanTools | pan-genomic data | [www.bif.wur.nl](http://www.bif.wur.nl) | Neo4j | 10.1093/bioinformatics/btw455 |
| LION LBD | A literature-based discovery system that enables researchers to navigate published information and supports hypothesis generation and testing | [lbd.lionproject.net](http://lbd.lionproject.net) | Neo4j | 10.1093/bioinformatics/bty845 |
| KnetMiner | KnetMiner is an integrated, intelligent, interactive gene network discovery platform that supports scientists explore and understand the biological stories of complex traits and diseases across species. It is also n example of application of KGs, based on FAIR principles. | knetminer.org; knetminer.com/ | Neo4j | 10.1111/pbi.13583 |
| GeNNet | GeNNet: platform for unifying scientific workflows and graph databases for transcriptome data analysis | [github.com/quelopes/GeNNet](https://github.com/quelopes/GeNNet) | Neo4j | 10.7717/peerj.3509 |
| STON | STON: tool to store SBGN networks in Neo4j databases | [sourceforge.net/projects/ston/](https://sourceforge.net/projects/ston/) | Neo4j | 10.1186/s12859-016-1394-x |
| GeneDiseaseRepositioning | An integrated Data Driven Approach to Drug Repositioning Using Gene-Disease Associations | [bitbucket.org/ncl-intbio/genediseaserepositioning/src/master/](https://bitbucket.org/ncl-intbio/genediseaserepositioning/src/master/) | Neo4j | 10.1371/journal.pone.0155811 |
| miTALOS | A tool for tissue specific pathway analysis of miRNAs | [mips.helmholtz-muenchen.de/mitalos](http://mips.helmholtz-muenchen.de/mitalos) | Neo4j | 10.1371/journal.pone.0151771 |
| cyNeo4j | cyNeo4j: connecting Neo4j and Cytoscape | [github.com/gsummer/cyneo4j-parent](https://github.com/gsummer/cyneo4j-parent) | Neo4j | 10.1093/bioinformatics/btv460 |
| FlyBrainLab | FlyBrainLab: an open-source computing platform for exploring and visualizing fly brain circuits datasets | [flybrainlab.fruitflybrain.org/](https://flybrainlab.fruitflybrain.org/) | OrientDB | 10.7554/eLife.62362 |
| JaNet | A tool for 3D exploration and visualization of lifespan data | [sysbio.uni-ulm.de/software/janet/](https://sysbio.uni-ulm.de/software/janet/) | Neo4j | 10.1186/s12859-018-2393-x |
| PanTools | A tool for the Inference of homologs in large eukaryotic pan-proteomes | [github.com/sheikhizadeh/pantools](https://github.com/sheikhizadeh/pantools) | Neo4j | 10.1186/s12859-018-2362-4 |
| ADR-graph | Knowledge-based machine learning tool to predict adverse drug reactions | [github.com/KHP-Informatics/ADR-graph](https://github.com/KHP-Informatics/ADR-graph) | Neo4j | 10.1038/s41598-017-16674-x |
| LifeWatchGreece | A set of data services for discovering biodiversity data | [metacatalogue.portal.lifewatchgreece.eu/](https://metacatalogue.portal.lifewatchgreece.eu/) | Virtuoso (RDF) | 10.3897/BDJ.4.e8443 |
| Open Tree of Life | Synthesis of phylogeny and taxonomy into a comprehensive tree of life | [tree.opentreeoflife.org](https://tree.opentreeoflife.org) | Neo4j | 10.1073/pnas.1423041112 |
| Matchmaker Exchange API | A tool for for Human disease gene discovery | [mme.monarchinitiative.org:9000](https://mme.monarchinitiative.org:9000) | Neo4j | 10.1002/humu.22857 |
| BioTCM-SE | An instantiated ontology for the integration of modern biology and traditional Chinese medicine concepts and data, and a query platform for this ontology | [www.biotcm.org/biotcm-se/indexpage/index.html](http://www.biotcm.org/biotcm-se/indexpage/index.html) | Virtuoso (RDF) | 10.1155/2014/957231 |
| MyTaxa | MyTaxa: an advanced taxonomic classifier for genomic and metagenomic sequences | [enve-omics.ce.gatech.edu/MyTaxa/](http://enve-omics.ce.gatech.edu/MyTaxa/) | Neo4j | 10.1093/nar/gku169 |
| NeXO | A gene ontology built computationally from gene networks backed by a database and visualization platform | [www.nexontology.org/](http://www.nexontology.org/) | Neo4j | 10.1093/nar/gkt1192 |
| Treemachine | Analyzing and Synthesizing Phylogenies Using Tree Alignment Graphs | [github.com/OpenTreeOfLife/treemachine](https://github.com/OpenTreeOfLife/treemachine) | Neo4j | 10.1371/journal.pcbi.1003223 |
| KGEV | framework for construction and visual exploration of KGs, two use cases explore a Covid KG and and the Human Phenotype Ontology | [covid19nlp.wglab.org/](http://covid19nlp.wglab.org/) | Neo4j | 10.1186/s12911-022-01848-z |
| GRIMM | human stem cell transplantation; graph traversal strategies to explore matching between patient and donor | [github.com/nmdp-bioinformatics/grimm](https://github.com/nmdp-bioinformatics/grimm) | Neo4j | 10.1093/bioinformatics/btz050 |
| BioGraph | Integrates multiple reources towards exploration of microRNA in breast cancer | biograph.pa.icar.cnr.it. | Neo4j; OrientDB | 10.1186/s12918-018-0616-4 |
| Recon2Neo4j | Integration of the Recon2 human metabolism reconstruction into Neo4j | [github.com/ibalaur/MetabolicFramework](https://github.com/ibalaur/MetabolicFramework) | Neo4j | 10.1093/bioinformatics/btw731 |
| HitWalker2 | it facilitates visual exploration of the existing HitWalker package developed for the translational research | [github.com/biodev/HitWalker2](https://github.com/biodev/HitWalker2) | Neo4j | 10.1093/bioinformatics/btv739 |
| PyBEL | It is a software package to manage the BEL format and to provides converters between BEL and several formats (including the neo4j format) | [github.com/pybel](https://github.com/pybel) | Neo4j | 10.1093/bioinformatics/btx660 |
| Metabox | Metabox represents a toolbox for metabolomics analysis (following integration with other omics: proteomics/ transcriptomics) | [kwanjeeraw.github.io/metabox/](http://kwanjeeraw.github.io/metabox/) | Neo4j | 10.1371/journal.pone.0171046 |
| COVID-19 Knowledge Graph | A COVID-19 KG using Virtuoso and integrating molecules and their interactions | research.bioinformatics.udel.edu/covid19kg/.; research.bioinformatics.udel.edu/covid19kg/ | Virtuoso (RDF) | 10.1093/bioinformatics/btab694 |
| BioDWH2 | BioDWH2: an automated graph-based data warehouse and mapping tool; collection of dedicated tools; data warehouse, mapping tool | [github.com/BioDWH2](https://github.com/BioDWH2) | GraphQL; Neo4j | 10.1515/jib-2020-0033 |
| GraphOmics | The GraphOmics is a user-friendly KG to explore and integrate multiple omics datasets (transcriptomics, proteomics and metabolomics) and support hypothesis generation. Integration on top of Reactome's GDB | [graphomics.glasgowcompbio.org/](https://graphomics.glasgowcompbio.org/) | Neo4j | 10.1186/s12859-021-04500-1 |
| WebProv | This is a neo4j-based system for semantic connection among systems biology models; Relating simulation studies by provenance—Developing a family of Wnt signaling models | [github.com/SFB-ELAINE/WebProv](https://github.com/SFB-ELAINE/WebProv) | Neo4j | 10.1371/journal.pcbi.1009227 |
| ROBOKOP | A tool for KGs exploration | [robokop.renci.org/](http://robokop.renci.org/) | Neo4j | 10.1093/bioinformatics/btz604 |
| SemNet | A tool for converting a database of semantic triples for indexed abstracts in PubMed into a semantic inference network and biomedical concept graph in Neo4j | [github.com/pathology-dynamics/semnet](https://github.com/pathology-dynamics/semnet) | Neo4j | 10.3389/fbioe.2019.00156 |
| Genet | A method to transform genetic circuit designs into networks | [github.com/intbio-ncl/genet2.git](https://github.com/intbio-ncl/genet2.git) | Neo4j | 10.1021/acssynbio.2c00255 |
| AnthraxKP | Anthrax Knowledge Portal includes a KG of genes, diseases, chemicals, species, vaccines, proteins related to Anthrax from biomedical literature, and an Anthrax Ontology | [139.224.212.120:18095/](http://139.224.212.120:18095/) | Neo4j | 10.1093/database/baac037 |
| OpenKG-COVID19 | A linked data set including 10 COVID-19 KGs, connected at both the schema and data levels. | [pkubase.gstore.cn](http://pkubase.gstore.cn) | gStore (RDF) | 10.2196/37215 |
| PubmedKG | A definition of semantic relations between 4 biomedical entity types (variants, genes, diseases, chemicals) extracted from >33mio pubmed abstracts | [www.pubmedkb.cc/](https://www.pubmedkb.cc/) | Neo4j | 10.1093/nar/gkac310 |
| SCKan KG | SCKan KG combines ApiNATOMY connectivity models and ApiNATOMY TOO map; aim: classify, navigate and search for semantically-linked metadata of multimodal experimental datasets for a number of cross-scale, cross-disciplinary projects. | SCKan: github.com/SciCrunch/sparc-curation/blob/master/docs/sckan/README.org, Apinatomy: github.com/SciCrunch/sparc-curation/blob/master/docs/apinatomy.org | Blazegraph (RDF); Neo4j | 10.3389/fphys.2022.795303 |
| pmTR database | Identification of true germline alleles enriched with complete TR allele sequences and their frequencies across 26 different human populations, profiled by “1000 Genomes data”; pmTR DB profiles the TR germline alleles for all four TR loci (TRA, TRB, TRG and TRD) | [pmtrig.lumc.nl/](https://pmtrig.lumc.nl/) | Neo4j | 10.1038/s41435-022-00171-x |
| HiMoRNA (Histone-Modifying RNA) database | HiMoRNA integrates a large amount of multi-omics data to characterize the effects of lncRNA on epigenetic modifications and gene expression | github.com/lab-medvedeva/himorna-frontend (platform frontend) and github.com/lab-medvedeva/himorna-backend | Neo4j | 10.3390/ncrna8010018 |
| CKG | CKG is an open-source platform currently comprising close to 20 million nodes and 220 million relationships that represent relevant experimental data, public databases and literature | github.com/MannLabs/CKG; documentation found at: CKG.readthedocs.io/en/latest/advanced_features/ckg-notebooks.html | Neo4j | 10.1038/s41587-021-01145-6 |
| ResearchMaps | A free, open-source web application that allows biologists to create research maps, graph-based representations of empirical evidence and hypothetical assertions found in research articles, reviews, and other sources | [researchmaps.org/](http://researchmaps.org/) | Neo4j | 10.1371/journal.pone.0195271 |
| MELODI | A literature mining platform that can identify mechanistic pathways between biomedical concepts | [www.melodi.biocompute.org.uk/](http://www.melodi.biocompute.org.uk/) | Neo4j | 10.1093/ije/dyx251 |
| SATORI | An integrative search and visual exploration interface for the exploration of biomedical data repositories | [satori.refinery-platform.org/](http://satori.refinery-platform.org/) | Neo4j | 10.1093/bioinformatics/btx739 |
| Graffinity | Visualizing Connectivity in Large Graphs with neuroscience in mind | [github.com/visdesignlab/graffinity](https://github.com/visdesignlab/graffinity) | Neo4j | 10.1111/cgf.13184 |
| Pathfinder | Introducing a technique for visual analysis of paths in graphs | [demo.caleydo.org/pathfinder/](http://demo.caleydo.org/pathfinder/) | Neo4j | 10.1111/cgf.12883 |
| ClustOmics | Multi-omics clustering approach; a generic consensus clustering tool used in the context of cancer subtyping | [github.com/galadrielbriere/ClustOmics](https://github.com/galadrielbriere/ClustOmics) | Neo4j | 10.1186/s12859-021-04279-1 |
| Mako | Software tool that converts microbiome data and networks into a graph database and visualises query results, thus allowing users without programming knowledge to carry out network-based queries | [github.com/ramellose/mako/](https://github.com/ramellose/mako/) | Neo4j | 10.1038/s41592-021-01335-9 |
| gcCov | A method for mapping data from publicly available genomics and publication resources to RDF (server to publish linked open data (LOD)implemented) | [nmdc.cn/gccov/](https://nmdc.cn/gccov/) | RDF | 10.1002/mlf2.12008 |
| COVID-19 Knowledge Graph | A COVID-19 KG integrating multimodal data (e.g. proteins, genes, chemicals and biological processes) | bikmi.covid19-knowledgespace.de/; github.com/covid19kg; bikmi.covid19-knowledgespace.de | OrientDB | 10.1093/bioinformatics/btaa834 |
| StonPy | A python-based library enabling storing and exploring modelcular maps available in the SBGN standard format in Neo4j | [github.com/adrienrougny/stonpy](https://github.com/adrienrougny/stonpy) | Neo4j | 10.1093/bioinformatics/btad100 |
| Graph4Med | A tool for visualising and analysis of patient cohorts (heath records and NGD data), also allowing full transformation of relational DB into Neo4J | [graph4med.cs.uni-frankfurt.de/](http://graph4med.cs.uni-frankfurt.de/) | Neo4j | 10.1186/s12859-022-05092-0 |
| PhageClouds | A genomic network graph of phage genomic sequences and their intergenomic distances to compare sequences | [phageclouds.dk/](https://phageclouds.dk/) | Neo4j | 10.1089/phage.2021.0008 |
| neuPrint | Integrates networks of neurons and synapses in a graph for analysis of electron microscopy connectomes | github.com/connectome-neuprint; neuprint.janelia.org/ | Neo4j | 10.3389/fninf.2022.896292 |
| Fragment Graph DataBase (FGDB) | A graph database of ligand fragments from the Protein Data Bank | [biochimica3.bio.uniroma3.it/fragments-web/](http://biochimica3.bio.uniroma3.it/fragments-web/) | Neo4j | 10.1093/database/baac044 |
| SynLethDB 2.0 | A new version of SynLethDB with new synthetic lethality gene pairs. A nice overview of the tool's architecture. It includes a case study of BRCA1 (breast cancer gene). Version 1 was build on relational DB. | [synlethdb.sist.shanghaitech.edu.cn/v2](https://synlethdb.sist.shanghaitech.edu.cn/v2) | Neo4j | 10.1093/database/baac030 |
| Knowledge Beacon API | Access to and comparison of several GDB resources is provided via a Swagger 2.0 API | [github.com/NCATS-Tangerine/translator-knowledge-beacon](https://github.com/NCATS-Tangerine/translator-knowledge-beacon) |  | 10.1371/journal.pone.0231916 |

## **Table S**6: List of COVID related resources available in the form of a GDB

| **Resource** | **Content** | **Accessible at** | **GDB** | **Publication** |
| --- | --- | --- | --- | --- |
| SciGraph | A presentation of the Monarch platform, which offers access to integrated biological data across different species | [github.com/SciGraph/SciGraph](https://github.com/SciGraph/SciGraph) | Neo4j | 10.1093/nar/gkz997 |
| KGEV | framework for construction and visual exploration of KGs, two use cases explore a Covid KG and and the Human Phenotype Ontology | [covid19nlp.wglab.org/](http://covid19nlp.wglab.org/) | Neo4j | 10.1186/s12911-022-01848-z |
| KG-COVID-19 | KG-COVID-19, a framework to build COVID-19 knowledge graphs | github.com/Knowledge-Graph-Hub/kg-covid-19/wiki; kg-hub-rdf.berkeleybop.io/blazegraph/#query | Blazegraph (RDF) | 10.1016/j.patter.2020.100155 |
| IntAct | IntAct GDB; molecular interaction data from the IntAct resource | [www.ebi.ac.uk/intact/download](https://www.ebi.ac.uk/intact/download); github.com/intact-portal | Neo4j | 10.1093/nar/gkab1006 |
| COVID-19 Knowledge Graph | A COVID-19 KG using Virtuoso and integrating molecules and their interactions | [research.bioinformatics.udel.edu/covid19kg/](https://research.bioinformatics.udel.edu/covid19kg/); research.bioinformatics.udel.edu/covid19kg/ | Virtuoso (RDF) | 10.1093/bioinformatics/btab694 |
| CovidGraph | A COVID-19 KG integrating biomedical data, papers, patents, clinical trials and systems biology models | [healthecco.org/covidgraph/](https://healthecco.org/covidgraph/) | Neo4j | 10.1093/bioinformatics/btac592 |
| CovidPubGraph | A KG of scientific publications about COVID-19 based on CORD-19 including NER, entity linking and link discovery | [covid-19ds.data.dice-research.org/sparql](https://covid-19ds.data.dice-research.org/sparql); github.com/dice-group/COVID19DS | RDF | 10.1038/s41597-022-01298-2 |
| OpenKG-COVID19 | A linked data set including 10 COVID-19 KGs, connected at both the schema and data levels. | [pkubase.gstore.cn](http://pkubase.gstore.cn) | gStore (RDF) | 10.2196/37215 |
| CovidGraph | A review of the current landscape of KGs for COVID-19 | [covidgraph.org/](https://covidgraph.org/) | Neo4j | 10.3390/jpm11040300 |
| gcCov | A method for mapping data from publicly available genomics and publication resources to RDF (server to publish linked open data (LOD)implemented) | [nmdc.cn/gccov/](https://nmdc.cn/gccov/) | RDF | 10.1002/mlf2.12008 |
| COVID-19 Knowledge Graph | A COVID-19 KG integrating multimodal data (e.g. proteins, genes, chemicals and biological processes) | bikmi.covid19-knowledgespace.de/; [github.com/covid19kg](https://github.com/covid19kg); bikmi.covid19-knowledgespace.de | OrientDB | 10.1093/bioinformatics/btaa834 |
| Neo4COVID19 | A workflow that integrates a COVID-19 specific host-host, host-pathogen, drug-target interaction network | [aspire.covid19.ncats.io:7473](https://aspire.covid19.ncats.io:7473) | Neo4j | 10.1021/acs.jcim.1c00431 |

## **Table S**7: List of systems biology primary resources available in the form of a GDB

| **Resource** | **Content** | **Accessible at** | **GDB** | **Publication** |
| --- | --- | --- | --- | --- |
| Plant Reactome | a knowledgebase and resource for comparative pathway analysis | [plantreactome.gramene.org/index.phplang=en](https://plantreactome.gramene.org/index.phplang=en) | Neo4j | 10.1093/nar/gkz996 |
| Reactome GDB | The GDB (neo4j) version of the Reactome KB | [reactome.org](https://reactome.org) | Neo4j | 10.1093/nar/gkx1132; 10.1371/journal.pcbi.1005968 |

## **Table S**8: List of systems biology integrated resources available in the form of a GDB

| **Resource** | **Content** | **Accessible at** | **GDB** | **Publication** | **Updates** |
| --- | --- | --- | --- | --- | --- |
| PDBe Aggregated API | A KG that integrates the content of the well-established Protein Data Bank resource | gitlab.ebi.ac.uk/pdbe-kb/services/pdbe-graph-api; pdbe.org/graph-api | Neo4j | 10.1093/bioinformatics/btab424 | Weekly |
| SmartGraph | A network pharmacology platform | [smartgraph.ncats.io/](https://smartgraph.ncats.io/) | Neo4j | 10.1186/s13321-020-0409-9 | Active |
| BioMedical Evidence Graph (BMEG) | Includes Pathway Commons data; Cancer; Gene expression and mutation data with drug-response experiments, pathway information databases, and literature-derived associations | [bmeg.io](http://bmeg.io) | Custom | 10.1200/CCI.19.00110 |  |
| GREG (The Gene Regulation Graph Database) | Multiple resources used | [www.moralab.science/GREG/](http://www.moralab.science/GREG/) | Neo4j | 10.1093/database/baz162 |  |
| BioKNO | Biological Knowledge Network Ontology (BioKNO), a lightweight ontology for the represention of biological-related knowledge networks | [github.com/Rothamsted/bioknet-onto](https://github.com/Rothamsted/bioknet-onto) | Neo4j | 10.1515/jib-2018-0023 |  |
| Hetionet (Project Rephetio) | A resource integrated millions of biomedical studies | [neo4j.het.io](http://neo4j.het.io) | Neo4j | 10.7554/eLife.26726 |  |
| EpiGeNet | A KG for colorectal cancer | [github.com/ibalaur/EpiGeNet](https://github.com/ibalaur/EpiGeNet) | Neo4j | 10.1089/cmb.2016.0095 |  |
| EffectorK | A comprehensive resource to mine protein interactions in the Arabidopsis proteome | [www.effectork.org/](http://www.effectork.org/) | Neo4j | 10.1111/mpp.12965 |  |
| Project Repethio (as a use case; published in a different paper) | Connections among drugs and diseases | [think-lab.github.io/p/rephetio/](https://think-lab.github.io/p/rephetio/) | Neo4j | 10.1021/acs.jmedchem.9b01989 |  |
| SciGraph | A presentation of the Monarch platform, which offers access to integrated biological data across different species | [github.com/SciGraph/SciGraph](https://github.com/SciGraph/SciGraph) | Neo4j | 10.1093/nar/gkz997 |  |
| SEAweb | An integrated resource for the analysis and visualization of sRNA expression data | [sea.ims.bio/](http://sea.ims.bio/) | Neo4j | 10.1093/nar/gkz869 | Active |
| PDBe-KB | A resource offering gunctional annotations of macromolecular structure data, contained in the Protein Data Bank (PDB) | [www.ebi.ac.uk/pdbe/pdbe-kb/](https://www.ebi.ac.uk/pdbe/pdbe-kb/) | Neo4j | 10.1093/nar/gkz853 | Active |
| Alliance of Genome Resources (Alliance) | A presentation of the Alliance of Genome Resource, which offers access to model organisms data and to the GO | [www.alliancegenome.org/](https://www.alliancegenome.org/) | Neo4j | 10.1093/nar/gkz813 | Active |
| DisGeNET-RDF | An integrated resource of gene-disease association | [www.disgenet.org/rdf](https://www.disgenet.org/rdf) | Virtuoso (RDF) | 10.1093/bioinformatics/btw214 | Active |
| KaBOB | 18 databases; Knowledge Base Of Biomedicine | [github.com/drlivingston/kabob](https://github.com/drlivingston/kabob) | AllegroGraph; Virtuoso (RDF) | 10.1186/s12859-015-0559-3 |  |
| EpiGraphDB | A resource focusing on biomedical and epidemiological relationships and providing an analytical framework also. | epigraphdb.org/; epigraphdb.org | Neo4j | 10.1093/bioinformatics/btaa961 | Active |
| graphd | Multiomics data from the Cancer Genome Atlas and imaging data | [github.com/ishworthapa/graphd](https://github.com/ishworthapa/graphd) | Neo4j | 10.1089/cmb.2020.0231 |  |
| COMBAT-TB-NeoDB | An integrated resource of M.tb 'omics' data | [github.com/COMBAT-TB](https://github.com/COMBAT-TB) | Neo4j | 10.1093/bioinformatics/btz658 |  |
| biochem4j | A resource integrating various external primary resources of biochemical data | [biochem4j.org](http://biochem4j.org) | Neo4j | 10.1371/journal.pone.0179130 |  |
| GeNNet | GeNNet: platform for unifying scientific workflows and graph databases for transcriptome data analysis | [github.com/quelopes/GeNNet](https://github.com/quelopes/GeNNet) | Neo4j | 10.7717/peerj.3509 |  |
| GeneDiseaseRepositioning | An integrated Data Driven Approach to Drug Repositioning Using Gene-Disease Associations | [bitbucket.org/ncl-intbio/genediseaserepositioning/src/master/](https://bitbucket.org/ncl-intbio/genediseaserepositioning/src/master/) | Neo4j | 10.1371/journal.pone.0155811 |  |
| masymos | A GDB prototype to store systems biology mathematical models (SBML, CellML), simulations (SED-ML) and annotations (ontologies) | [sems.uni-rostock.de/projects/masymos/](https://sems.uni-rostock.de/projects/masymos/) | Neo4j | 10.1093/database/bau130 |  |
| FlyBrainLab | FlyBrainLab: an open-source computing platform for exploring and visualizing fly brain circuits datasets | [flybrainlab.fruitflybrain.org/](https://flybrainlab.fruitflybrain.org/) | OrientDB | 10.7554/eLife.62362 |  |
| Neuprint | A connectome and analysis of the adult Drosophila central brain. | [neuprint.janelia.org](https://neuprint.janelia.org) | Neo4j | 10.7554/eLife.57443 |  |
| MicrobeFDT | A resource which clusters chemically similar drug and food compounds and links these compounds to microbial enzymes and known toxicities | [github.com/kellylab/microbeFDT-neo4j](https://github.com/kellylab/microbeFDT-neo4j) | Neo4j | 10.7554/eLife.42866 |  |
| PanTools | A tool for the Inference of homologs in large eukaryotic pan-proteomes | [github.com/sheikhizadeh/pantools](https://github.com/sheikhizadeh/pantools) | Neo4j | 10.1186/s12859-018-2362-4 |  |
| ADR-graph | Knowledge-based machine learning tool to predict adverse drug reactions | [github.com/KHP-Informatics/ADR-graph](https://github.com/KHP-Informatics/ADR-graph) | Neo4j | 10.1038/s41598-017-16674-x |  |
| LifeWatchGreece | A set of data services for discovering biodiversity data | [metacatalogue.portal.lifewatchgreece.eu/](https://metacatalogue.portal.lifewatchgreece.eu/) | Virtuoso (RDF) | 10.3897/BDJ.4.e8443 |  |
| Open Tree of Life | Synthesis of phylogeny and taxonomy into a comprehensive tree of life | [tree.opentreeoflife.org](https://tree.opentreeoflife.org) | Neo4j | 10.1073/pnas.1423041112 |  |
| Matchmaker Exchange API | A tool for for Human disease gene discovery | [mme.monarchinitiative.org:9000](https://mme.monarchinitiative.org:9000) | Neo4j | 10.1002/humu.22857 |  |
| GORouter | An RDF model which encodes heterogeneous original data in a uniform RDF format, creates additional ontology mappings between GO terms, and introduces a set of inference rulebases | [www.scbit.org/gorouter/](http://www.scbit.org/gorouter/) | Oracle 10g NDM (RDF) | 10.1186/1471-2105-9-S1-S6 |  |
| KGEV | framework for construction and visual exploration of KGs, two use cases explore a Covid KG and and the Human Phenotype Ontology | [covid19nlp.wglab.org/](http://covid19nlp.wglab.org/) | Neo4j | 10.1186/s12911-022-01848-z |  |
| NoName | This is a large biomedical knowledge based for rare diseases including a comprehensive set of 3,819,623 nodes and 84,223,681 inter-relationships, based on 34 biomedical resources. While not provided in a landing page, the crediatials are the default in neo4j community edition (user: neo4j; pwd: neo4j) | [disease.ncats.io](https://disease.ncats.io) | Neo4j | 10.1186/s13326-020-00232-y |  |
| starPepDB | It integrates bioactive peptide extracted from a large set of dedicated resources | [mobiosd-hub.com/starpep/](http://mobiosd-hub.com/starpep/) | Neo4j | 10.1093/bioinformatics/btz260 |  |
| GenCoNet | Various molecular interrelationships; starting point: essential hypertension and bronchial asthma | [genconet.kalis-amts.de](https://genconet.kalis-amts.de) | Neo4j | 10.1515/jib-2018-0049 |  |
| HRGRN | HRGRN, an integrative database of Arabidopsis molecular networks | [plantgrn.noble.org/hrgrn/](http://plantgrn.noble.org/hrgrn/) | Neo4j | 10.1093/pcp/pcv200 |  |
| OTAR interactome | Integrated resource + graph-based analytics | ftp://ftp.ebi.ac.uk/pub/databases/intact/various/ot_graphdb/current) | Neo4j | 10.1038/s41588-023-01327-9 |  |
| GeneFriends | gene co-expression database | [www.genefriends.org](http://www.genefriends.org) | Neo4j | 10.1093/nar/gkac1031 | Active |
| TWAS Atlas | manually colected gene–trait associations, single nucleotide polymorphism (SNP)–gene associations | [ngdc.cncb.ac.cn/twas/](https://ngdc.cncb.ac.cn/twas/) | Neo4j | 10.1093/nar/gkac821 |  |
| RTX-KG2 | creates a knowledge graph that integrates data from multiple sources in a standardized way. | [github.com/RTXteam/RTX-KG2](https://github.com/RTXteam/RTX-KG2) | Neo4j | 10.1186/s12859-022-04932-3 |  |
| PDBe-KB | describes the GDB components for the Protein Data Bank in Europe | [www.ebi.ac.uk/pdbe/pdbe-kb/](https://www.ebi.ac.uk/pdbe/pdbe-kb/) | Neo4j | 10.1002/pro.4439 |  |
| NeDRexDB | Describes an integrated framework that supports network-based analytics for disease module identification and drug repurposing | [neo4j.nedrex.net/](http://neo4j.nedrex.net/) | Neo4j | 10.1038/s41467-021-27138-2 |  |
| Synthetic Lethality Knowledge Graph (SLKG) | Cancer; Synthetic lethality, Synthetic dosage lethality | www.slkg.net:81/search; www.slkg.net/ | Neo4j | 10.1038/s41467-021-21544-2 |  |
| COVID-KOP (based on ROBOKOP) | Covid-19 knowledge graph construction | covidkop.renci.org/ (not working); github.com/NCATS-Gamma/robokop | Neo4j | 10.1093/bioinformatics/btaa718 |  |
| KG-COVID-19 | KG-COVID-19, a framework to build COVID-19 knowledge graphs | github.com/Knowledge-Graph-Hub/kg-covid-19/wiki; kg-hub-rdf.berkeleybop.io/blazegraph/#query | Blazegraph (RDF) | 10.1016/j.patter.2020.100155 |  |
| SEARCHIN | An analytical pipeline that integrates several existent functionalities and was developed to prioritize interactions between ligands and receptors in amyotrophic lateral sclerosis | [github.com/Califano-lab/SEARCHIN](https://github.com/Califano-lab/SEARCHIN) | Neo4j | 10.1038/s41467-020-19177-y |  |
| Virtuoso | authors create an aggregated DB of various life science features using invididual initially RDF-DBs | [integbio.jp/rdf/](https://integbio.jp/rdf/) | Virtuoso (RDF) | 10.1093/database/bay123 | Active |
| NoName | resource for exploration of virus - genome interactions in colon (cancer) | [github.com/SchlossLab/Hannigan_CRCVirome_mBio_2018](https://github.com/SchlossLab/Hannigan_CRCVirome_mBio_2018) | Neo4j | 10.1128/mBio.02248-18 |  |
| Chemotext | A resource connecting publications on drug-target-disease | [chemotext.mml.unc.edu/](http://chemotext.mml.unc.edu/) | Neo4j | 10.1021/acs.jcim.7b00589 |  |
| Tabloid Proteome | Integrates content on inferred protein-protein association | [iomics.ugent.be/tabloidproteome](http://iomics.ugent.be/tabloidproteome) | Neo4j | 10.1093/nar/gkx930 |  |
| ncRNA-DB | it contains ncRNAs, genes, and associated diseases and inter-relationships | [ncrnadb.scienze.univr.it/ncrnadb/](http://ncrnadb.scienze.univr.it/ncrnadb/) | OrientDB | 10.3389/fbioe.2014.00069 |  |
| BioModels Linked Dataset | Authors developed the BioModels Linked Dataset to store an RDF representation of all BioModels models | [www.ebi.ac.uk/rdf/services/sparql](https://www.ebi.ac.uk/rdf/services/sparql) | Virtuoso (RDF) | 10.1186/s12918-014-0091-5 | Active |
| NetworkAnalyst | A network-based framrwork for integration, analysis and visualisation of gene expression profiling | [www.networkanalyst.ca/](https://www.networkanalyst.ca/) | Neo4j | 10.1093/nar/gku443 | Active (on the website) |
| HCLS KB | Demonstration of several semantic web query federation approaches to integrate diverse types of data in neurology | www.corporate-semantic-web.de/hcls.html; hcls.deri.org/sparql | AllegroGraph; Virtuoso (RDF) | 10.1186/1471-2105-10-S10-S10 |  |
| IntAct | IntAct GDB; molecular interaction data from the IntAct resource | www.ebi.ac.uk/intact/download; github.com/intact-portal | Neo4j | 10.1093/nar/gkab1006 | Active |
| PDBe-KB | Structural information; annotations derived from or relevant to the Protein Data Bank (PDB); Protein Data Bank in Europe – Knowledge Base | pdbe-kb.org; pdbe-kb.org/graph-download; pdbe-kb.org/graph-download | Neo4j | 10.1093/nar/gkab988 | Active |
| BioSamples | A sample metadata database at EMBL-EBI following FAIR principles; Descriptions and metadata about biological samples | [www.ebi.ac.uk/biosamples](http://www.ebi.ac.uk/biosamples) | Neo4j | 10.1093/nar/gkab1046 |  |
| Inxight Drugs; NCATS Inxight Drugs | An extension of the G-SRS codebase; Drug database: 125 036 product ingredients, including 2566 US approved drugs, 6242 marketed drugs, and 9684 investigational drugs | drugs.ncats.io; drugs.ncats.io/ | Neo4j | 10.1093/nar/gkab918 | Active |
| COVID-19 Knowledge Graph | A COVID-19 KG using Virtuoso and integrating molecules and their interactions | research.bioinformatics.udel.edu/covid19kg/.; research.bioinformatics.udel.edu/covid19kg/ | Virtuoso (RDF) | 10.1093/bioinformatics/btab694 |  |
| BioDWH2 | BioDWH2: an automated graph-based data warehouse and mapping tool; collection of dedicated tools; data warehouse, mapping tool | [github.com/BioDWH2](https://github.com/BioDWH2) | GraphQL; Neo4j | 10.1515/jib-2020-0033 | Active |
| GraphOmics | The GraphOmics is a user-friendly KG to explore and integrate multiple omics datasets (transcriptomics, proteomics and metabolomics) and support hypothesis generation. Integration on top of Reactome's GDB | [graphomics.glasgowcompbio.org/](https://graphomics.glasgowcompbio.org/) | Neo4j | 10.1186/s12859-021-04500-1 |  |
| Metabolic Atlas | A KB for relationships between potential biomarkers - disease - (no) known treatment; Resource of genome scale metabolic networks of model animals | [metabolicatlas.org/](https://metabolicatlas.org/) | Neo4j | 10.1073/pnas.2102344118 |  |
| RPGeNet v2.0 | A tool to visualize the interactome network of visual disorder genes | [compgen.bio.ub.edu/RPGeNet](https://compgen.bio.ub.edu/RPGeNet) | Neo4j | 10.1093/database/baz120 |  |
| MaSyMoS | Proposing a workflow that identifies frequent structural patterns in biochemical reaction networks | [github.com/MaSyMoS](https://github.com/MaSyMoS) | Neo4j | 10.1093/database/bay051 |  |
| ODG | A tool for creating customized databases that utilize published genomics data integrated with experimental data which can be queried using a flexible graph database | [github.com/jguhlin/odg](https://github.com/jguhlin/odg) | Neo4j | 10.1186/s12859-017-1777-7 |  |
| SPOKE | Knowledge graph for precision medicine built from integrating 41 databases and based on ontologies | [spoke.rbvi.ucsf.edu/](https://spoke.rbvi.ucsf.edu/) | Neo4j | 10.1093/bioinformatics/btad080 | Active |
| CovidGraph | A COVID-19 KG integrating biomedical data, papers, patents, clinical trials and systems biology models | [healthecco.org/covidgraph/](https://healthecco.org/covidgraph/) | Neo4j | 10.1093/bioinformatics/btac592 |  |
| CovidPubGraph | A KG of scientific publications about COVID-19 based on CORD-19 including NER, entity linking and link discovery | covid-19ds.data.dice-research.org/sparql; github.com/dice-group/COVID19DS | RDF | 10.1038/s41597-022-01298-2 | Paper states biweekly updates |
| AnthraxKP | Anthrax Knowledge Portal includes a KG of genes, diseases, chemicals, species, vaccines, proteins related to Anthrax from biomedical literature, and an Anthrax Ontology | [139.224.212.120:18095/](http://139.224.212.120:18095/) | Neo4j | 10.1093/database/baac037 |  |
| OpenKG-COVID19 | A linked data set including 10 COVID-19 KGs, connected at both the schema and data levels. | [pkubase.gstore.cn](http://pkubase.gstore.cn) | gStore (RDF) | 10.2196/37215 |  |
| HerbKG | A KG that bridges herbal and molecular medicine, included entities are herbs, chemicals extracted from the herbs, genes that are affected by the chemicals, and diseases treated by herbs due to the functions of genes. | [github.com/FeiYee/HerbKG](https://github.com/FeiYee/HerbKG) | Neo4j | 10.3389/fgene.2022.799349 |  |
| ImmuneData | The data discovery system ImmuneData provides integrated access to five immunology data repositories: ImmPort, ImmuneSpace, ITN TrialShare, ImmGen and IEDB (uniform metadata schema) | [www.immunedata.org/](http://www.immunedata.org/) | Neo4j | 10.1093/database/baac003 |  |
| COVID-KOP | Creating a new knowledgebase integrating an existing biomedical knowledge graph (ROBOKOP) with information from recent biomedical literature on COVID-19 | [covidkop.renci.org/](https://covidkop.renci.org/) | Neo4j | 10.26434/chemrxiv.12462623 |  |
| ResearchMaps | A free, open-source web application that allows biologists to create research maps, graph-based representations of empirical evidence and hypothetical assertions found in research articles, reviews, and other sources | [researchmaps.org/](http://researchmaps.org/) | Neo4j | 10.1371/journal.pone.0195271 |  |
| The Protein API | A tool that provides searching and programmatic access to protein and associated genomics data such as curated protein sequence positional annotations from UniProtKB, as well as mapped variation and proteomics data from large scale data sources | [www.ebi.ac.uk/proteins/api/doc](http://www.ebi.ac.uk/proteins/api/doc) | Neo4j | 10.1093/nar/gkx237 | Active |
| ChemRDF | A database system that adresses certain shortcomings of RDF databases that make their use restrictive or challenging for common users, demonstrated on the ChEBI database | [bioinfo.uochb.cas.cz/projects/chemRDF](https://bioinfo.uochb.cas.cz/projects/chemRDF) | Virtuoso (RDF) | 10.1186/s13321-016-0144-4 |  |
| ClustOmics | Multi-omics clustering approach; a generic consensus clustering tool used in the context of cancer subtyping | [github.com/galadrielbriere/ClustOmics](https://github.com/galadrielbriere/ClustOmics) | Neo4j | 10.1186/s12859-021-04279-1 |  |
| LinkedImm | A linked data graph approach to integration of immunological data; diverse types of vaccine response measurement data from the NIH/NIAID ImmPort data repository, pathway data from Reactome, influenza virus strains from WHO, and taxonomic data from NCBI Taxon | [linkedimm.org](http://linkedimm.org) | Neo4j | 10.1109/bibm47256.2019.8982986 |  |
| ABCkb | The Aliment to Bodily Condition knowledgebase (ABCkb): a database connecting plants and human health. Neo4j is used to store the knowledge graph. | [abckb.charlotte.edu](https://abckb.charlotte.edu) | Neo4j | 10.1186/s13104-021-05835-x |  |
| COVID-19 Knowledge Graph | A COVID-19 KG integrating multimodal data (e.g. proteins, genes, chemicals and biological processes) | bikmi.covid19-knowledgespace.de/; github.com/covid19kg; bikmi.covid19-knowledgespace.de | OrientDB | 10.1093/bioinformatics/btaa834 | Active |
| PlagueKD | A GDB for plague-related knowledge also integrating PubMed-extracted information | [39.104.28.169:18095/](http://39.104.28.169:18095/) | Neo4j | 10.1093/database/baac100 |  |
| GenomicKB | A KG for human genome, epigenome, transcriptome, 4D nucleome that integrates genomic datasets and annotations | [gkb.dcmb.med.umich.edu/](https://gkb.dcmb.med.umich.edu/) | Neo4j | 10.1093/nar/gkac957 |  |
| ROBOKOP | An exploration of metal-implant related adverse outcome pathways (toxicity mechanisms) | [robokopkg.renci.org/browser/](http://robokopkg.renci.org/browser/) | Neo4j | 10.1016/j.yrtph.2022.105277 |  |
| PhageClouds | A genomic network graph of phage genomic sequences and their intergenomic distances to compare sequences | [phageclouds.dk/](https://phageclouds.dk/) | Neo4j | 10.1089/phage.2021.0008 |  |
| PharMeBINet | Knowledge graph that integrates biomedical pharmacological databases and Hetionet | [pharmebi.net](https://pharmebi.net) | Neo4j | 10.1038/s41597-022-01510-3 |  |
| Fragment Graph DataBase (FGDB) | A graph database of ligand fragments from the Protein Data Bank | [biochimica3.bio.uniroma3.it/fragments-web/](http://biochimica3.bio.uniroma3.it/fragments-web/) | Neo4j | 10.1093/database/baac044 |  |
| BioTAGME | A system that combines an entity-annotation framework (TAGME) with a network-based inference methodology (DT-Hybrid) | [biotagme.eu](https://biotagme.eu) | Neo4j | 10.3389/fgene.2022.855739 | Paper mentions "periodical updates" |
| SynLethDB 2.0 | A new version of SynLethDB with new synthetic lethality gene pairs. A nice overview of the tool's architecture. It includes a case study of BRCA1 (breast cancer gene). Version 1 was build on relational DB. | [synlethdb.sist.shanghaitech.edu.cn/v2](https://synlethdb.sist.shanghaitech.edu.cn/v2) | Neo4j | 10.1093/database/baac030 |  |
| ERMer (E. coli Regulation Miner) | A framework to explore regulatory landscape of E. coli with interactions obtained from published databases, such as BiGG, STITCH, STRING and RegulonDB | [ermer.biodesign.ac.cn/](https://ermer.biodesign.ac.cn/) | Amazon Neptune | 10.1093/nar/gkac288 |  |
| Neo4COVID19 | A workflow that integrates a COVID-19 specific host-host, host-pathogen, drug-target interaction network | [aspire.covid19.ncats.io:7473](https://aspire.covid19.ncats.io:7473) | Neo4j | 10.1021/acs.jcim.1c00431 |  |
| Ortho_KB | A knowledge graph framework to utilize translational approaches in plant research, specifically grain legumes | ortholegkb.versailles.inrae.fr/browser/; forgemia.inra.fr/geapsi/ecp-paper/ortholegkb_data | Neo4j | 10.3389/frai.2023.1191122 |  |
| iBKH | A comprehensive BKG called the integrative Biomedical Knowledge Hub (iBKH) by harmonizing and integrating information from diverse biomedical resources | [ibkh.ai/](http://ibkh.ai/) | Neo4j | 10.1016/j.isci.2023.106460 |  |
| CyanoMapDB | A database providing cyanobacterial PPIs with experimental evidence | [www.cyanomapdb.msbio.pro/](http://www.cyanomapdb.msbio.pro/) | Neo4j | 10.1093/plphys/kiac594 |  |

# Supplementary methods

The table below offers a comparison of the four popular open-source GDBs: Neo4, ArangoDB, OrientDB, and Virtuoso. Currently, Neo4j is the most widely used open-source GDB tool; however, there is also a comparable number of works that use the Virtuoso, ArangoDB, and OrientDB multi-model databases (DB), which combine different types of non-relational DBs simultaneously, for example allowing the use of document-oriented data to include queries through publications linking them to the biological objects they describe.

| **Database** | **Database type** | **Models included** | **Query language** | **Release year** | **Implementation** | **Data Scheme** | **SQL support** |
| --- | --- | --- | --- | --- | --- | --- | --- |
| Neo4j | Graph | Graph | Cypher | 2007 | Java | Schema-free and schema-optional | Yes* |
| ArangoDB | Multi-model | Document; Graph; Key-value; Search engine | AQL | 2012 | C++ | Schema-free | No |
| OrientDB | Multi-model | Document; Graph; Key-value; | Gremlin | 2010 | Java | Schema-free | No |
| Virtuoso | Multi-model | Document; Graph; Native XML; Relational; RDF; Search engine | SPARQL | 1998 | C | SQL - Standard relational schema; RDF - Quad/Triple;  XML - DTD, XML schema | Yes |

*The Neo4j Enterprise distribution includes the BI Connector, a JDBC-compatible interface, that allows executing SQL queries over a Neo4j resource.

## PubMed and PMC search queries

PubMed (pubmed.ncbi.nlm.nih.gov) and PubMed Central (PMC; ncbi.nlm.nih.gov/pmc) were searched for relevant publications with a cut-off date of 31/03/2023 using the key words "graph database" or "graph databases", including the top 16 popular graph databases according to DB-Engines (as given in Table 1) such as Neo4j, Azure Cosmos, ArangoDB, etc. The key words used for the search were chosen in order to maximise the specificity of the results in relation to graph database publications (thus, to minimise the number of accidental matches). For example, for the Virtuoso graph database, it is “Openlink AND Virtuoso”, since simply searching for “Virtuoso” returned instances where the key word was matched to the family name of authors in PubMed/PMC instead of the graph database technology. Other examples are “Apache AND Giraph” and “Fauna AND graph database". For Stardog, we used “Stardog AND graph database" because it is one of the multi-model DBs and is often used as an RDF store and not specifically as a GDB. For unique names we directly used the names of the DBs such as “Neo4j” or "Azure Cosmos DB" or “ArangoDB”.

Specifically, we used Search Query #1, since the search in PubMed found key phrases in titles and abstracts, results containing only the abstract term “graph database” were also included, as they could have specific DBs used in the work, but not mentioned in the title. This search query covered most publications where GDBs were mentioned.

| **Search Query #1:** "graph database" OR "graph databases" OR Neo4j OR "Azure Cosmos" OR ArangoDB OR OrientDB OR (Openlink AND Virtuoso) OR (Ontotext AND GraphDB) OR JanusGraph OR "Amazon Neptune" OR (Stardog AND "graph database") OR TigerGraph OR FaunaDB OR (Fauna AND "graph database") OR AllegroGraph OR (Dgraph AND "graph database") OR (Giraph AND "graph database") OR "Nebula Graph" OR Memgraph |
| --- |

Then, we further used Search query #2 to search specifically for mentions of specific GDBs (including Neo4j, ArangoDB, etc.) in full-text publications in PMC in order to prioritise the publication list: a publication presenting a direct use of a specific GDB technology was given higher priority than a publication mentioning only the GDB technology.

| **Search Query #2:** Neo4j OR "Azure Cosmos" OR ArangoDB OR OrientDB OR (Openlink AND Virtuoso) OR (Ontotext AND GraphDB) OR JanusGraph OR "Amazon Neptune" OR (Stardog AND "graph database") OR TigerGraph OR FaunaDB OR (Fauna AND "graph database") OR AllegroGraph OR (Dgraph AND "graph database") OR (Giraph AND "graph database") OR "Nebula Graph" OR Memgraph |
| --- |

Search query #2, focusing on specific GDBs, acts as a refinement criteria for our analysis: for full-text PMC publications, we considered only those mentioning the top 16 GDB technologies/ approaches.

## Python script to create the results table

We developed a Python script to merge the results of these search queries via PMID, PMCID, and DOI, available in CSV files, thus consolidating a single table with all relevant information. The Python script and its output is available at github.com/ilyamazein/gdbreview.

## Manual review: criteria for inclusion/exclusion and subdivision into categories

After removing duplicated publications (n=146), we aggregated a list of n=681 publications to be screened for this review. Each shortlisted publication was manually annotated by two reviewers. Several important inclusion/ exclusion criteria followed during the manual review allowed accelerating the process, minimising time and effort for further review of a full-length publication:

1. First, we considered only publications with the full text accessible to us (open or via our institutes). We also considered only publications with the text provided in English.
2. Second, we checked and confirmed that a certain GDB technology was not simply mentioned but was actually applied in the work described. Publications that only mention but not use a GDB were removed. We also removed preprints or conference posters.
3. Third, we removed publications describing integrated resources that were not available at the location mentioned in the publication nor provided a repository for the source code.
4. We selected only publications where a GDB was used in bioinformatics or systems biology context. For the COVID-19 knowledge bases, we extended the inclusion to systems biomedicine as the COVID-19 resources themselves can be classified as interdisciplinary, incorporating medical and other data.

We grouped these according to their content in several major categories. The table below includes the respective definitions and the section in this review addressing them in detail. Note: a few SOFTWARE publications were assigned to multiple categories given that details were provided also for the methodological approach. For example, one may be considered as a SOFTWARE and an ONTOLOGY publication given detailed description of both the integrated knowledge base as well as the network-based method used for the analysis.

Further, we manually annotated each publication with details on the GDB technology used in the publication (e.g., Neo4j, Virtuoso, OrientDB), the name, and the url in the case of integrated resources/softwares/tools as well as maintenance information if available. We also briefly summarised the content of the publication, and extremely important, we annotated reasons on including/ excluding the respective publication in the current review.

| **Category name** | **Definition** | **Name of the section in this review addressing details** |
| --- | --- | --- |
| REVIEW | if the publication was a review dedicated to the application of GDB approaches in bioinformatics and systems biology; | Information from these reviews was integrated in the main text, when needed. |
| METHOD | if the publication presented a method developed using a GDB approach for addressing a specific problem or question in systems biology; | Mainly presented in the Tools section. |
| SOFTWARE | if a tool developed using a GDB approach for research was described. In this case, the publications were refined based on the availability of the resource itself to be queried or of the development code (e.g. github repository), and only those providing supporting urls for these points were retained for full text consideration. Supplementary FIle Software includes details on the software/ tools regarding the name, availability, and updates; | Software tools were described mainly in the Tools section. |
| PRIMARY | if the publication presented a GDB version of original resources. For example, Reactome graph database. These PRIMARY resources can be used for developing more complex resources that include multiple sources (see INTEGRATED below); | Mostly presented in the “Pathway biology“ section. |
| INTEGRATED | if the publication described an GDB resource developed by integrating multiple DBs from systems biology (e.g. resources on pathways, biomarkers and drug-targets); | This is the most extended category of publications included in the review. . Mostly presented in the “Pathway biology“ and the “Systems biology use-case: COVID-19 resources” sections. |
| ONTOLOGY | if a GDB approach to describe and facilitate access to the terms of an ontology was presented. | Mainly presented in the “Ontologies” section. |
| OTHERS | if a GDB approach was used for purposes different than above, such as integration of web semantic data or of medical data etc. | Publications marked as OTHERS were removed during the full text revision, being considered out of scope. |
